# Investigating associations between rural-to-urban migration and cardiometabolic disease in Malawi: a population-level study

**DOI:** 10.1093/ije/dyz198

**Published:** 2019-10-11

**Authors:** Felix P Chilunga, Crispin Musicha, Terence Tafatatha, Steffen Geis, Moffat J Nyirenda, Amelia C Crampin, Alison J Price

**Affiliations:** 1 Malawi Epidemiology and Intervention Research Unit (MEIRU), Lilongwe and Karonga, Malawi; 2 Institute for Medical Microbiology and Illnesses, Philipps University of Marburg, Marburg, Germany; 3 Department of Infectious Disease Epidemiology, London School of Hygiene & Tropical Medicine, London, UK; 4 MRC/UVRI & LSHTM Uganda Research Unit, Kampala, Uganda

**Keywords:** Migration, urbanization, cardiovascular risk factors, diabetes, obesity, Africa

## Abstract

**Background:**

The extent to which rural-to-urban migration affects risk for cardiometabolic diseases (CMD) in Africa is not well understood. We investigated prevalence and risk for obesity, diabetes, hypertension and precursor conditions by migration status.

**Methods:**

In a cross-sectional survey in Malawi (February 2013–March 2017), 13 903 rural, 9929 rural-to-urban migrant and 6741 urban residents (≥18 years old) participated. We interviewed participants, measured blood pressure and collected anthropometric data and fasting blood samples to estimate population prevalences and odds ratios, using negative binomial regression, for CMD, by migration status. In a sub-cohort of 131 rural–urban siblings-sets, migration-associated CMD risk was explored using conditional Poisson regression.

**Results:**

In rural, rural-to-urban migrant and urban residents, prevalence estimates were; 8.9, 20.9 and 15.2% in men and 25.4, 43.9 and 39.3% in women for overweight/obesity; 1.4, 2.9 and 1.9% in men and 1.5, 2.8 and 1.7% in women for diabetes; and 13.4, 18.8 and 12.2% in men and 13.7, 15.8 and 10.2% in women for hypertension. Rural-to-urban migrants had the greatest risk for hypertension (adjusted relative risk for men 1.18; 95% confidence interval 1.04–1.34 and women 1.17: 95% confidence interval 1.05–1.29) and were the most screened, diagnosed and treated for CMD, compared with urban residents. Within sibling sets, rural-to-urban migrant siblings had a higher risk for overweight and pre-hypertension, with no evidence for differences by duration of stay.

**Conclusions:**

Rural-to-urban migration is associated with increased CMD risk in Malawi. In a poor country experiencing rapid urbanization, interventions for the prevention and management of CMD, which reach migrant populations, are needed.

**Key Messages**

- There is higher prevalence of overweight/obesity, hypertension and diabetes in rural-to-urban migrants than in either urban or rural residents.
- Higher prevalences of obesity, hypertension and their precursor states (overweight and pre-hypertension) were observed in urban migrant siblings compared with their rural non-migrant siblings.
- Rural-to-urban migrants report higher access to screening, diagnosis and treatment for hypertension and diabetes than either urban or rural residents.
- Interventions to prevent and manage cardiometabolic disease need to reach the growing migrant population in rapidly urbanizing sub-Saharan Africa.

## Introduction

Urbanization is shaping epidemiological and demographic transition in sub-Saharan Africa (SSA).^1^ In Malawi, one of the poorest countries in SSA, 84% of the 18 million population live in rural areas, yet internal net rural-to-urban migration, predominantly for economic reasons, has been increasing steadily at 4.1% per annum.^2^^,^^3^ Whereas urbanization associated with economic growth has beneficial effects on raising standards of living, there are also potentially deleterious effects of urbanization on health.^4^ Adoption of a sedentary lifestyle, smoking and alcohol consumption, increased consumption of energy-dense foods and psychosocial stress have been shown to contribute to higher cardiometabolic disease (CMD) prevalence in urban compared with rural areas.^4^^,^^5^

In Malawi, the burden of CMD is high. A 2009 national STEPwise approach to Surveillance (STEPS) prevalence survey reported 27% overweight/obesity, 33% hypertension and 6% diabetes, with higher prevalences of all conditions in urban compared with rural adults.^6^ Data on migrant populations were not available. A more recent population-based survey comparing urban to rural adults (*n* = 29 000) showed higher prevalences of overweight/obesity (40 vs 20%), hypertension (23 vs 15%) and diabetes (5 vs 2%) in urban compared with rural residents.^7^

Evidence for an effect of migration on risk for hypertension, diabetes and obesity is emerging from middle-income countries (MIC)^8–10^ but findings from low-income countries (LIC) of SSA are few.^11^^,^^12^ Current evidence suggests that rural-to-urban migrants experience a higher risk of obesity and diabetes than rural residents and, although this risk increases with duration of stay, it remains lower than that observed in urban residents.^13^ Findings for the association between rural-to-urban migration and hypertension are inconsistent.^11^^,^^14^

Malawi presents a unique context in which to study the relationship between rural-to-urban migration and CMD in SSA. The rate of rural-to-urban migration is high and lifestyles vary substantively, with higher prevalence of early-life adverse factors—maternal undernutrition, prenatal injurious agents and early-childhood undernutrition—in rural compared with urban residents, which may impact CMD development in later life.^15^^,^^16^

We, therefore, hypothesized that changes in behaviour, lifestyle, healthcare and psychosocial stress in adulthood combined with adverse conditions earlier in the life course would contribute to higher CMD risk in rural-to-urban migrants compared with either urban or rural residents. We used population-level cross-sectional data to investigate associations between rural-to-urban migration and obesity, diabetes, hypertension and their precursor states. To minimize the impact of residual confounding on estimates of effect for rural-to-urban migration, we investigated these associations within a sibling-sets sub-study of rural-born siblings with at least one urban migrant sibling.^17^

## Methods

### Study setting and population

Between February 2013 and March 2017, we conducted population-based non-communicable disease surveys to quantify the burden and distribution of risk factors in rural and urban Malawi. Detailed study methods have been published elsewhere.^18^ In brief, 13 903 rural and 16 670 urban Malawian men and women aged ≥18 years were recruited in rural Northern Karonga district and in Lilongwe, the capital city. In Karonga, we recruited participants from the Karonga Health and Demographic Surveillance Site (HDSS) and defined these participants as rural residents.^19^ In Lilongwe, we conducted the urban survey in Area-25—a high-density residential area.^18^ Urban Lilongwe residents who were born outside of the major cities (Blantyre or Lilongwe) were defined as rural-to-urban migrants and all others as urban residents.

### Sibling sub-study

We nested a retrospective cohort study of siblings within these two study sites to understand in more detail the effect of migration on risk for CMDs within families.^18^ At the rural site, we identified study participants with siblings known to have migrated to the urban area (Lilongwe city) using migration data from the HDSS database. After obtaining consent from the rural-non-migrant sibling, we made initial contact with the rural-to-urban migrant sibling by telephone, inviting them to participate in the urban-based surveys.

### Ethical consideration

The National Health Sciences Research Committee of Malawi approved the study. We translated patient information sheets, consent and questionnaire material into appropriate local languages. Participants provided written informed consent before commencing an interview.

### Measurements

We modified the World Health Organization STEPwise approach to chronic-disease risk-factor surveillance (WHO STEPS) instrument and questions from the Hyderabad study to meet local needs and used standardized methods for anthropometric measurements and venepuncture sample collection in both study sites.^6^^,^^20^ We defined pre-hypertension as systolic blood pressure (SBP) between 120 and 139 mmHg and/or a diastolic blood pressure (DBP) between 80 and 89mmHg and hypertension as a SBP ≥140mmHg and/or a DBP ≥90mmHg or self-report of current anti-hypertensive medication. We defined impaired fasting glucose (IFG) as fasting blood glucose (FBG) between 6.1 and 6.9 mmol/l and diabetes as fasting blood glucose ≥7.0 mmol/l, or on regular medication for diabetes, or a previous self-reported diagnosis of diabetes by a health professional. We defined overweight as body mass index (BMI) between 25 and 29 kg/m^2^ and obesity as BMI ≥30 kg/m^2^. We defined waist-to-hip ratio (WHR) as high when ≥0.95 for men and ≥0.85 for women.^21^ We defined multimorbidity as the presence of two or more of hypertension, diabetes and obesity.

We categorized education according to the highest level reached in primary (standard 1–5 or 6–8), secondary and university education. For the sibling sub-study, we categorized education broadly into completed or not completed primary school. Occupation data were collected in pre-coded categories and further categorized into: not working, housework, farming/fishing, self-employed and employed. A student category was also used for the sibling sub-study. We used locally determined estimated monetary values of assets to create a cumulative asset value from which we generated proxy wealth scores, categorized into fifths across the total study population.^7^

To calculate levels of physical activity, we used the Global Physical Activity Questionnaire (GPAQ).^22^ We generated average metabolic equivalent of task (MET) data per day by combining self-reported duration (minutes) and intensity (pre-coded activities, grouped into high or low exertion and sedentary) of physical activity in the previous week (work and leisure). This was further categorized according to whether participants met the World Health Organization (WHO) recommendations of at least 600 Total Physical Activity MET minutes per week. We categorized smoking into not current (never and former) and current smokers, where former smokers were participants who had stopped within the preceding 6 months at the time of data collection. We categorized alcohol consumption as: not taken any in last year or taken any in the last year. We asked participants about the number of teaspoons of sugar added to each cup of tea/coffee (range 0–10), average number of cups per day (range 0–10) and usual number of pre-sweetened drinks (carbonated and local brands; range 0–30) in order to calculate the average daily sugar consumption (teaspoon equivalents, in drinks). To categorize sugar consumption in drinks, we used WHO guidelines: <6 or ≥6 teaspoons per day. We used information on the household size, reported frequency of household purchases of a standard measure of plain salt (equivalent to a 50-g bag of salt, which was shown during the interview) to estimate daily average per-capita home consumption, categorized as <2.5, 2.5–5, 5.1–7.5 and >7.5 gm/day.

### Statistical analysis

We investigated differences in socio-demographic and health-related behavioural risk factors in rural residents, rural-to-urban migrants and urban residents separately by sex. We applied age-specific rates of overweight/obesity, hypertension and diabetes to the WHO standard population to generate age-standardized population prevalence estimates for comparison between sites and with external populations.^23^ We used a negative binomial regression model with a log-link function to calculate risk ratios for overweight/obesity, hypertension, diabetes and multimorbidity, adjusting for age and sex. To account for potential clustering (as recruitment included all household adults and family members share factors such as socio-economic status and diet), we calculated robust standard errors. If FBG was not available and there was no self-reported prior diabetes diagnosis, we excluded participants from the diabetes-prevalence calculation. In multivariable models, we adjusted for age, sex, level of education, occupation, wealth quintile, smoking, alcohol consumption and physical activity, as appropriate. For adjustment variables, missing values represented <5% of the data in every variable. We used Chi-squared likelihood ratio tests to assess for heterogeneity in the association of migration status and health outcomes (diabetes, hypertension, overweight/obesity) by sex.

In rural–urban sibling sets, where at least one sibling was rural and one was urban-dwelling, we used conditional Poisson regression to calculate relative risks for several health states including IFG, diabetes, pre-hypertension, hypertension, overweight and obesity, with adjustment for potential confounders. Rural–urban sibling sets shared both parents but were not matched on age or sex (1:1). For sibling sets of three or more, we matched each rural sibling to all their urban siblings of any age or sex. For urban migrant siblings, we investigated the association of length of urban residency (<5 years, ≥5 years) and the health outcomes using logistic regression. We performed all analyses using Stata version 14.0 (2015; Stata 14.0 Statistical Software, College Station, TX, USA).

## Results

### Demographic characteristics

A total of 40 173 individuals were approached (15 806 rural, 24 367 urban) and 30 573 (76%) enrolled in the population-level survey; 13 903 were rural residents (born and dwelling), 9929 were rural-to-urban migrants and 6741 were urban residents (born and dwelling) (Supplementary Figure 1, available as Supplementary data at *IJE* online). The majority of participants were women (61.8%). Mean (SD) age was highest in rural residents (38 ± 16 years). Rural residents were less educated and poorer than rural-to-urban migrants and urban residents. Women had lower levels of education compared with men. Rural residents were mostly subsistence farmers, a high proportion of rural-to-urban migrants were domestic workers and the majority of urban residents were not employed (students, retired, unemployed; Table 1). The most common reason for migration was seeking employment (43.4%; Supplementary Table 7, available as Supplementary data at *IJE* online).

**Table 1.** Baseline socio-demographic characteristics of population-survey participants by migration status

	Total			Men			Women		
	Karonga	Lilongwe		Karonga	Lilongwe		Karonga	Lilongwe	
	Rural (*n* = 13 903)	Urban (*n* = 6741)	Rural-to-urban migrant (*n* = 9929)	Rural (*n* = 5864)	Urban (*n* = 2348)	Rural-to-urban migrants (*n* = 3456)	Rural (*N* = 8039)	Urban (*N* = 4393)	Rural-to-urban migrant (*N* = 6473)
	*n*(%)	*n*(%)	*n*(%)	*n*(%)	*n*(%)	*n*(%)	*n*(%)	*n*(%)	*n*(%)
**Age group**									
18–24	3329 (23.9)	2614 (38.9)	2886 (27.1)	1493 (25.5)	1015 (43.2)	1039 (30.1)	1836 (22.8)	1599 (36.4)	1847 (28.5)
25–34	3761 (27.1)	2368 (35.1)	3303 (33.3)	1521 (24.9)	707 (30.1)	963 (27.9)	2240 (27.9)	1661 (37.8)	2340 (36.2)
35–44	2783 (20.0)	1030 (15.3)	1864 (18.9)	1169 (19.9)	369 (15.7)	706 (20.4)	1614 (20.1)	661 (15.1)	1158 (17.9)
45–54	1703 (12.3)	384 (5.7)	960 (9.7)	728 (12.4)	116 (4.9)	362 (10.5)	975 (12.1)	268 (6.1)	598 (9.2)
55–64	1069 (7.7)	190 (2.8)	526 (5.3)	422 (7.2)	72 (3.1)	211 (6.1)	647 (8.1)	118 (2.7)	315 (4.9)
65+	1258 (9.1)	155 (2.3)	390 (3.9)	531 (9.1)	69 (2.9)	175 (5.1)	727 (9.0)	86 (1.9)	215 (3.3)
Age mean (sd)^a^	37.9 (16.4)	30.3 (11.7)	33.8 (13.5)	37.8 (16.5)	30.1 (12.4)	34.8 (14.4)	38.1 (16.3)	30.4 (11.4)	33.3 (13.1)
**Wealth quintile**									
Poorest	3739 (26.9)	1013 (15.0)	1371 (13.8)	1391 (23.7)	281 (11.9)	381 (11.0)	2348 (29.2)	732 (16.7)	990 (15.3)
2	4077 (29.3)	954 (14.2)	1464 (14.7)	1772 (30.2)	298 (12.7)	478 (13.8)	2305 (28.7)	656 (14.9)	986 (15.2)
3	2962 (21.3)	1151 (17.1)	1700 (17.1)	1320 (22.5)	405 (17.3)	623 (18.0	1642 (20.4)	746 (16.9)	1077 (16.6)
4	2012 (14.5)	1709 (25.4)	2530 (25.5)	877 (14.9)	634 (27.0)	911 (26.4)	1135 (14.1)	1075 (24.5)	1619 (25.0)
Wealthiest	1113 (8.0)	1914 (28.4)	2864 (28.8)	504 (8.6)	730 (31.1)	1063 (30.8)	609 (7.6)	1184 (26.9)	1801 (27.8)
**Education**									
None	595 (4.3)	228 (3.4)	356 (3.6)	99 (1.7)	33 (1.4)	45 (1.3)	496 (6.2)	195 (4.4)	311 (4.8)
Standard 1–5	2004 (14.4)	450 (6.7)	880 (8.9)	635 (10.8)	96 (4.1)	176 (5.1)	1369 (17.1)	354 (8.1)	704 (10.9)
Standard 6–8	6520 (46.9)	1090 (16.2)	2212 (22.3)	2430 (41.4)	269 (11.5)	546 (15.8)	4090 (50.9)	821 (18.7)	1666 (25.7)
Secondary	4531 (32.6)	3707 (54.9)	4994 (50.3)	2525 (43.1)	1373 (58.5)	1965 (56.9)	2006 (24.9)	2334 (53.1)	3029 (46.8)
Tertiary	253 (1.8)	1266 (18.8)	1487 (14.9)	175 (2.9)	577 (24.6)	724 (20.9)	78 (0.9)	689 (15.7)	763 (11.8)
**Employment**									
Not working	1788 (12.9)	2130 (31.6)	2491 (25.1)	1038 (17.7)	992 (42.3)	1099 (31.8)	750 (9.3)	1138 (25.9)	1392 (21.5)
Housework	838 (6.0)	1883 (27.9)	3101 (31.2)	70 (1.2)	161 (6.9)	203 (5.9)	768 (9.6)	1722 (39.2)	2898 (44.8)
Farming/fishing	8658 (62.3)	45 (0.7)	42 (0.4)	3376 (57.6)	12 (0.5)	18 (0.5)	5282 (65.7)	33 (0.8)	24 (0.4)
Self-employed	1896 (13.6)	1251 (18.6)	1869 (18.8)	853 (14.6)	462 (19.7)	701 (20.3	1043 (12.9)	789 (17.9)	1168 (18.0)
Employed	723 (5.2)	1432 (21.2)	2426 (24.4)	527 (8.9)	721 (30.7)	1435 (41.5)	196 (2.4)	711 (16.2)	991 (15.3)

a *P* for difference in mean age by migration status (rural, urban, rural-to-urban migrants) was <0.0001 in total population, in males and in females.

A total of 231 rural siblings and 129 urban migrant siblings participated in the sibling sub-cohort study (Supplementary Figure 1, available as Supplementary data at *IJE* online). About half were women (50.3%) and the mean (SD) age was 31 ± 9 years. A total of 348 (96.7%) had completed primary-school education. The median length of stay in the urban area was 6.0 (IQR 3.0–9.5) years. The most common occupation for rural siblings was subsistence activities whereas most urban migrant siblings were employed (Table 2).

**Table 2.** Baseline socio-demographic and health-related characteristics of urban and rural siblings

	Total siblings	Urban resident siblings	Rural resident siblings
	N = 360	N = 129	N = 231
	*n*(%)	*n*(%)	*n*(%)
**Sex**			
Men	179 (49.7)	62 (48.1)	117 (50.6)
Women	181 (50.3)	67 (51.9)	114 (49.4)
Age; mean (sd)	31.1 (8.8)	30.7 (8.3)	31.2 (17.6)
**Education**			
Completed primary education	348 (96.7)	123 (95.4)	225 (97.4)
Not completed primary education	12 (3.3)	6 (4.7)	6 (2.6)
**Occupation**			
Student	52 (14.5)	25 (19.4)	27 (11.7)
Not working	16 (4.4)	7 (5.4)	9 (3.9)
Housework	30 (8.3)	16 (12.4)	14 (6.1)
Farming/fishing	124 (34.5)	4 (3.1)	120 (52.1)
Self-employed	68 (18.9)	22 (17.1)	46 (19.9)
Employed	70 (19.4)	55 (42.6)	15 (6.5)
**Body mass index kg/m^2^**			
<18	16 (4.4)	10 (4.7)	6 (4.3)
18–24.9	243 (67.5)	165 (60.7)	78 (71.4)
25–29.9	61 (16.9)	30 (24.0)	31 (12.9)
≥30	30 (8.3)	18 (9.3)	12 (7.8)
Unknown	10 (2.8)	8 (1.6)	2 (3.5)
**Waist-to-hip ratio; median (IQR)**	0.8 (0.8–0.9)	0.9 (0.8–0.9)	0.8 (0.8–0.9)
**Systolic blood pressure mmHg; median (IQR)^a^**	119.5 (110.8–129)	126.5 (116.5–135.5)	116.0 (110.5–124.0)
**Diastolic blood pressure mmHg; median (IQR)** ^a^	72.5 (66.5–79.0)	75.0 (69.5–81.0)	71.0 (65.5–76.5)
**Length of stay (years): median (IQR)**	6.0 (3.0–9.5)	6.0 (3.0–9.5)	
**Physical activity** ^b^			
Did not meet	2 (0.6)	1 (0.4)	1 (0.4)
Met recommended	358 (99.4)	230 (99.6)	128 (99.2)
**Smoking**			
Never	339 (94.2)	216 (93.5)	123 (95.5)
Former	5 (1.4)	1 (0.4)	4 (3.1)
Current	16 (4.4)	14 (6.1)	2 (1.4)
**Alcohol consumption**			
Not in last year	276 (76.7)	176 (76.2)	100 (77.5)
In last year	84 (23.3)	55 (23.8)	29 (22.5)
**Sugary drinks intake**			
<6 tsps/day	124 (34.4)	11 (8.5)	113 (48.9)
≥6 tsps/day	192 (53.3)	99 (76.7)	93 (40.3)
Unknown	44 (12.2)	19 (14.7)	25 (10.8)

a *P* for difference in mean systolic blood pressure and diastolic blood pressure among rural siblings and urban siblings was <0.0001.

b Metabolic equivalents of task (MET) according to World Health Organization (WHO) criteria. Recommended MET of at least 600 per week.

### Lifestyle risk factors

For the population-level survey, site- and gender-specific crude prevalence estimates for modifiable lifestyle risk factors are shown in Table 3. Rural-dwelling men had the highest reported levels of sugar consumption. Both rural-dwelling men and women were more likely to be living in a household with high usage of plain salt. Urban-dwelling men were more likely to be alcohol and tobacco consumers. Almost all participants in the three groups met the WHO physical-activity recommendations.

**Table 3.** Unadjusted prevalence of body mass index, blood pressure, diabetes and lifestyle risk-factor categories by migration status

	Total			Men			Women		
	Karonga	Lilongwe		Karonga	Lilongwe		Karonga	Lilongwe	
	Rural	Urban	Rural-to-urban migrants	Rural	Urban	Rural-to-urban migrants	Rural	Urban	Rural-to-urban migrants
	*N* = 13 903	*N* = 6741	*N* = 9929	*N* = 5864	*N* = 2348	*N* = 3456	*N* = 8039	*N* = 4393	*N* = 6473
**BMI^a^; kg/m^2^, *n*(%)**									
<18	707 (5.1)	240 (3.6)	255 (2.6)	350 (5.9)	110 (4.7)	129 (3.7)	357 (4.4)	130 (2.9)	126 (1.9)
18–24.9	10 087 (72.6)	4180 (62.1)	5770 (58.1)	4977 (85.1)	1880 (80.1)	2604 (75.4)	5110 (63.6)	2300 (52.4)	3166 (48.9)
25–29.9	1931 (13.9)	1332 (19.8)	2245 (22.6)	463 (7.9)	288 (12.3)	567 (16.4)	1468 (18.3)	1044 (23.8)	1678 (25.9)
30+	631 (4.5)	752 (11.2)	1318 (13.3)	59 (1.0)	69 (2.9)	155 (4.5)	572 (7.1)	683 (15.5)	1163 (18.0)
Unknown or pregnant	547 (3.9)	237 (3.5)	341 (3.4)	15 (0.3)	1 (0.0)	1 (0.0)	532 (6.6)	236 (5.4)	340 (5.3)
Mean^h^ BMI (sd); (kg/m^2^)	22.6 (3.8)	23.9 (4.9)	24.6 (4.9)	21.54 (2.7)	22.1 (3.3)	22.7 (3.7)	23.4 (4.3)	25.1 (5.3)	25.6 (5.3)
**WHR^b^, *n* (%)**									
Normal	8208 (59.0)	5329 (79.1)	7321 (73.7)	4671 (79.7)	2055 (87.5)	2786 (80.6)	3537 (44.0)	3274 (74.5)	4535 (70.1)
High	5194 (37.4)	1179 (17.5)	2273 (22.9)	1193 (20.3)	293 (12.5)	670 (19.4)	4001 (49.8)	886 (20.2)	1603 (24.8)
Unknown/pregnant	501 (3.6)	233 (3.5)	335 (3.4)	0.0	0.0	0.0	501 (6.2)	233 (5.3)	335 (5.2)
Mean^h^ WHR (sd)	0.9 (0.1)	0.8 (0.1)	0.8 (0.1)	0.9 (0.1)	0.8 (0.1)	0.8 (0.1)	0.9 (0.1)	0.8 (0.1)	0.8 (0.1)
**BP^c^; mmHg, *n* (%)**									
Normal	12 008 (86.4)	6005 (89.1)	8251 (83.1)	5074 (86.5)	2061 (87.8)	2808 (81.3)	6934 (86.3)	3944 (89.8)	5443 (84.1)
Mild hypertension	944 (6.8)	363 (5.4)	733 (7.4)	492 (8.2)	172 (7.3)	340 (9.8)	452 (5.6)	191 (4.3)	393 (6.1)
Moderate	314 (2.3)	126 (1.9)	260 (2.6)	135 (2.1)	56 (2.4)	107 (3.1)	179 (2.2)	70 (1.5)	153 (2.3)
Severe	121 (0.9)	47 (0.7))	135 (1.4)	33 (0.4)	13 (0.6)	49 (1.4)	88 (1.1)	34 (0.8)	86 (1.2)
On medication	509 (3.7)	197 (2.9)	545 (5.5)	127 (2.6)	45 (1.9)	152 (4.5)	382 (4.7)	152 (3.5)	393 (6.1)
Unknown	7 (0.1)	3 (0.0)	5 (0.1)	3 (0.1)	1 (0.0)	0.0	4 (0.1)	2 (0.1)	5 (0.1)
Mean^h^ systolic BP (sd); mmHg^2^	121.1 (17.8)	121.9 (15.5)	124.5 (18.1)	124.2 (15.6)	126.1 (14.0)	128.8 (16.7)	118.9 (19.1)	119.6 (15.7)	122.2 (18.3)
Mean^h^ diastolic BP (sd); mmHg^2^	72.8 (10.4)	72.6 (10.7)	74.6 (11.5)	73.0 (10.3)	72.3 (10.9)	75.1 (11.9)	72.6 (10.6)	72.8 (10.6)	74.3 (11.2)
**FBG^d^; mmol/L, *n* (%)**									
<6.1	11 771 (84.7)	4936 (73, 2)	7367 (74.2)	4869 (83.0)	1657 (70.6)	2441 (70.6)	6902 (85.9)	3279 (74.6)	4926 (76.1)
6.1–6.9	174 (1.3)	62 (0.9)	145 (1.5)	71 (1.2)	16 (0.7)	53 (1.5)	103 (1.3)	46 (1.1)	92 (1.4)
≥7.0	98 (0.7)	49 (0.7)	103 (1.1)	43 (0.7)	19 (0.7)	33 (0.9)	55 (0.6)	30 (0.7)	70 (1.1)
On medication	110 (0.8)	71 (1.1)	177 (1.8)	41 (0.7)	25 (1.0)	67 (1.9)	69 (0.9)	46 (1.1)	110 (1.7)
Unknown	1750 (12.6)	1623 (24.1)	2137 (21.5)	840 (14.3)	631 (26.9)	862 (24.9)	910 (11.3)	992 (22.6)	1275 (19.7)
Mean^h^ FBG (sd), mmol/L^2^	4.7 (1.2)	4.8 (1.2)	4.9 (1.4)	4.7 (1.2)	4.8 (1.2)	4.9 (1.3)	4.7 (1.2)	4.8 (1.1)	4.9 (1.4)
**Multimorbidity^e^, *n* (%)**									
None	9667 (69.5)	3924 (58.2)	5415 (54.5)	4276 (72.9)	1447 (61.6)	2018 (58.4)	5391 (67.1)	2477 (56.4)	3396 (52.5)
One	1727 (12.4)	857 (12.7)	1615 (16.3)	668 (11.4)	232 (9.9)	461 (13.3)	1059 (13.2)	625 (14.2)	1154 (17.8)
Two	283 (2.0)	175 (2.6)	460 (4.6)	64 (1.1)	38 (1.6)	115 (3.3)	219 (2.7)	137 (3.1)	345 (5.3)
Three	31 (0.2)	30 (0.5)	77 (0.8)	4 (0.1)	4 (0.2)	14 (0.4)	27 (0.3)	26 (0.6)	63 (0.9)
Unknown	2195 (15.8)	1755 (26.0)	2363 (23.8)	852 (14.5)	627 (26.7)	848 (24.5)	1343 (16.7)	1128 (25.7)	1515 (23.4)
**Physical activity: World Health Organization (WHO) recommendation^f^, *n* (%)**									
Did not meet	293 (2.1)	133 (1.9)	285 (2.9)	145 (2.5)	81 (3.4)	159 (4.6)	148 (1.8)	52 (1.2)	126 (1.9)
MET recommended	13 610 (97.9)	6608 (98.1)	9644 (97.1)	5719 (97.5)	2267 (96.6)	3297 (95.4)	7891 (98.2)	4341 (98.8)	6347 (98.1)
**Smoking, *n* (%)**									
Not current	12 826 (92.3)	6273 (93.1)	9379 (94.5)	4811 (82.0)	1913 (81.5)	2943 (85.2)	8015 (99.7)	4360 (99.3)	6436 (99.4)
Current	272 (1.9)	211 (3.1)	274 (2.8)	262 (4.5)	193 (8.2)	249 (7.2)	10 (0.1)	18 (0.4)	25 (0.4)
Unknown	805 (5.8)	257 (3.8)	276 (2.8)	791 (13.5)	242 (10.3)	264 (7.6)	14 (0.2)	15 (0.3)	12 (0.2)
**Alcohol consumption, *n* (%)**									
**Not in last year**	11 154 (80.2)	5396 (80.1)	8439 (84.9)	3404 (58.1)	1338 (56.9)	2305 (66.7	7750 (96.4)	4058 (92.4)	6134 (94.8)
In last year	2749 (19.8)	1345 (19.9)	1490 (15.1)	2460 (41.9)	1010 (43.1)	1151 (33.3)	289 (3.6)	335 (7.6)	339 (5.2)
**Sugary drinks intake, *n* (%)**									
**<6 tsps/day**	7225 (51.9)	3524 (52.3)	5134 (51.7)	2672 (45.6)	1148 (48.9)	1626 (47.1)	4553 (56.6)	2376 (54.1)	3508 (54.2)
≥6 tsps/day	5298 (38.1)	2288 (33.9)	3269 (32.9)	2766 (47.2)	869 (37.0)	1320 (38.2)	2532 (31.5)	1419 (32.3)	1949 (30.1)
Unknown	1380 (10.0)	929 (13.8)	1526 (15.4)	426 (7.3)	331 (14.1)	510 (14.8)	954 (11.8)	598 (13.6)	1016 (15.7)
**Salt intake^g^; gm/day, *n* (%)^h^**									
<2.5	2429 (17.7)	511 (7.6)	827 (8.3)	1277 (21.8)	252 (10.7)	365 (10.6)	1152 (14.3)	259 (5.9)	462 (7.1)
2.5–5	4028 (28.9)	2589 (38.4)	3789 (38.2)	1504 (25.7)	868 (36.9)	1259 (36.4)	2524 (31.4)	1721 (39.2)	2530 (39.1)
5.1–7.5	3192 (22.9)	1924 (28.5)	2743 (27.6)	1278 (21.8)	640 (27.3)	890 (25.8)	1914 (23.8)	1284 (29.2)	1853 (28.6)
>7.5	4039 (29.1)	1206 (17.9)	1882 (18.9)	1709 (29.1)	394 (16.8)	686 (19.9)	2330 (28.9)	812 (18.5)	1196 (18.5)
Unknown	215 (1.5)	511 (7.5)	688 (6.9)	96 (1.6)	194 (8.3)	256 (7.4)	119 (1.5)	317 (7.2)	432 (6.7)

a BMI (body mass index) defined according to WHO criteria: underweight (<18 kg/m^2^), normal (18–24.9 kg/m^2^), overweight (25–29.9 kg/m^2^) and obese (≥30 kg/m^2^).

b WHR (waist-to-hip ratio) defined according to WHO criteria. Central obesity defined as ≥0.95 for males and ≥0.85 for females.

c BP (blood pressure) defined according to WHO criteria. Hypertension is defined as systolic (SBP) ≥140 mmHg, diastolic (DBP) ≥90 mmHg or self-reported that currently using anti-hypertensive medication. Blood pressure categorized as normal (<130/80 mmHg), pre-hypertensive (131/81–139/89 mmHg), mild (140/90–159/99 mmHg), moderate (160/100–179/109 mmHg) and severe (>180/110 mmHg).

d FBG (fasting blood glucose) defined according to WHO criteria. Impaired fasting glucose (IFG) is defined as fasting blood glucose (FBG) between 6.1–6.9 mmol/l and diabetes as fasting blood glucose ≥7.0 mmol/l, or on regular medication for diabetes, or a previous self-reported diagnosis of diabetes by a health professional.

e Multimorbidity is defined as two or more of hypertension, diabetes and obesity.

f Physical activity was calculated using metabolic equivalents of task (MET) and categorized according to whether participants achieved the WHO recommendation of at least 600 MET per week. See Global Physical Activity Questionnaire analysis guide: https://www.who.int/ncds/surveillance/steps/resources/GPAQ_Analysis_Guide.pdf (accessed 14 June 2019).

g Reported household purchases of salt (whether used in cooking or added at the table).

h P for difference in mean BMI, WHR and systolic BP by migration status (rural, urban and rural-to-urban migrants) was <0.0001 in total population, in males and in females. *P* for difference in mean FBG was <0.0001 in total population and in females and 0.2 in males.

In the sibling-sets sub-study, urban migrant siblings were more likely to consume more sugary drinks compared with rural siblings. The proportion of participants who consumed alcohol in the preceding year was similar in rural and urban migrant siblings. Tobacco smoking was rare in both sibling groups. All siblings met the WHO physical-activity recommendation, regardless of migration status.

### Overweight and obesity

In the population study, results are presented separately in men and women due to statistically significant heterogeneity (*p* < 0.001) by sex in the association of migration status and cardiometabolic factors including overweight/obesity, hypertension and diabetes (Table 4). Crude prevalence of overweight/obesity in rural residents, rural-to-urban migrants and urban residents was 8.9, 20.9 and 15.2% for men and 25.4, 43.9 and 39.3% for women (Table 3). When standardized to the WHO world population, the overall prevalence of overweight/obesity was 19.9, 41.3 and 38.2% in rural, rural-to-urban migrants and urban residents, respectively (Supplementary Table 6, available as Supplementary data at *IJE* online). Despite lower mean BMI, rural men and women had the highest prevalence of high WHR (Table 3). In all groups, the prevalence of overweight/obesity increased with age until 50–59 years in women and 60–69 years in men (Supplementary Figure 2, available as Supplementary data at *IJE* online). Compared with urban residents, risk for overweight/obesity was reduced in rural men and women [adjusted relative risk (aRR) men 0.73, 95% confidence interval (CI) 0.63–0.86; aRR women 0.66, 95% CI 0.62–0.71] and modestly increased in the total rural-to-urban migrant population (aRR 1.05, 95% CI 1.03–1.11). The magnitude of risk for overweight/obesity increased with increasing education and wealth in rural, urban and rural-to-urban migrants (Supplementary Tables 1–3, available as Supplementary data at *IJE* online).

**Table 4.** Associations of migration status with cardiometabolic disorders and multimorbidity

	Total	Total age- and sex-adjusted	Total fully adjusted	Men	Men age- adjusted	Men fully adjusted	Women	Women age-adjusted	Women fully adjusted
	*n*(%)	RR^a^	RR^a^^,^^b^	*n*(%)	RR^a^	RR^a^^,^^b^	*n*(%)	RR^a^	RR^a^^,^^b^
Rural	13 903			5864			8039		
Urban	6741			2348			4393		
Rural-to-urban migrants	9929			3456			6473		
**Overweight/obesity^c^**									
Rural	2562(18.4))	0.52(0.49–0.55)	0.67(0.63–0.72)	522(8.9)	0.44(0.39–0.50)	0.73(0.63–0.86)	2040(25.4)	0.55(0.52–0.58)	0.66(0.62–0.71)
Urban	2084(30.9)	1	1	357(15.2)	1	1	1727(39.3)	1	1
Rural-to-urban migrants	3563(35.9)	1.07(1.03–1.12)	1.05(1.01–1.11)	722(20.9)	1.16(1.04–1.30)	1.09(0.98–1.22)	2841(43.9)	1.05(0.99–1.09)	1.05(0.99–1.08)
**Hypertension^d^**									
Rural	1888(13.6)	0.65(0.61–0.71)	0.79(0.71–0.87)	787(13.4)	0.69(0.61–0.79)	0.80(0.69–0.93)	1101(13.7)	0.61(0.55–0.68)	0.74(0.65–0.84)
Urban	733(10.9)	1	1	286(12.2)	1	1	447(10.2)	1	1
Rural-to urban migrants	1673(16.9)	1.20(1.10–1.29)	1.18(1.09–1.27)	648(18.8)	1.19(1.05–1.36)	1.18(1.04–1.34)	1025(15.8)	1.21(1.09–1.34)	1.17(1.05–1.29)
**Diabetes^e^**									
Rural	228(1.5)	0.39(0.31–0.49)	0.57(0.43–0.77)	84(1.4)	0.36(0.25–0.52)	0.44(0.29–0.68)	124(1.5)	0.40(0.30–0.55)	0.66(0.46–0.96)
Urban	120(1.8)	1	1	44 (1.9)	1	1	76(1.7)	1	1
Rural-to-urban migrants	281(2.8)	1.13(0.92–1.39)	1.07(0.88–1.33)	101(2.9)	1.07(0.76–1.53)	0.96(0.69–1.36)	180(2.8)	1.15(0.89–1.51)	1.12(0.87–1.46)
**Multimorbidity^f^**									
Rural	2041(2.3)	0.32(0.27–0.39)	0.63(0.51–0.78)	736(1.2)	0.28(0.19–0.42)	0.54(0.34–0.86)	1305(3.1)	0.34(0.28–0.41)	0.64(0.51–0.82)
Urban	1062(3.0)	1	1	274(1.8)	1	1	788(3.7)	1	1
Rural-to-urban migrants	215 (5.4)	1.22(1.04–1.43)	1.16(0.99–1.35)	590(3.7)	1.39(0.99–1.97)	1.23(0.88–1.72)	1562(6.3)	1.16(0.98–1.39)	1.11(0.93–1.31)

a RR are risk ratios (95% CI).

b Adjusted for age, sex, education, occupation, wealth status, smoking, alcohol consumption and physical activity.

c Overweight defined as body mass index (BMI) 25–29.9 kg/m^2^ and obesity defined as BMI ≥30 kg/m^2^.

d Hypertension is defined as systolic blood pressure ≥140 mmHg, diastolic blood pressure ≥90 mmHg, or self-reported that currently using anti-hypertensive medication.

e Diabetes mellitus (DM) defined as fasting blood glucose ≥7.0 mmol/L or current use of medication prescribed to treat diabetes mellitus or self-reported.

f Multimorbidity defined as the presence of two or more of hypertension, diabetes and obesity.

In the sibling-sets sub-study, urban migrant siblings had a higher risk of overweight/obesity compared with rural siblings after adjustment for confounding factors (aRR 2.06, 95% CI 1.03–4.12; Supplementary Table 5, available as Supplementary data at *IJE* online). However, there was no evidence for difference in risk by longer duration (≥5 vs <5 years) of urban stay (aRR 3.11, 95% CI 0.68–14.16).

### Blood pressure

Crude prevalence of hypertension in rural residents, rural-to-urban migrants and urban residents was 13.4, 18.8 and 12.2% for men and 13.7, 15.8 and 10.2% for women, respectively (Table 3). After WHO world-population age standardization, the prevalence of hypertension was 16.3, 26.7 and 23.4% in rural, rural-to-urban migrants and urban residents, respectively (Supplementary Table 6, available as Supplementary data at *IJE* online). In men and women, risk of hypertension was highest in rural-to-urban migrants compared with urban residents, after adjusting for confounders (aRR men 1.18, 95% CI 1.04–1.34; aRR women 1.17, 95% CI 1.05–1.29; Table 4). In women, greater wealth was associated with increased risk for hypertension in all study groups, whereas a higher education level was associated with increased risk for hypertension in urban and rural men but not rural-to-urban migrant men (Supplementary Tables 1–3, available as Supplementary data at *IJE* online). Of those eligible for screening (age >40 years and overweight/obese), rural-to-urban migrants were more likely to be screened, diagnosed and be on medication for hypertension than urban or rural residents (Supplementary Table 4, available as Supplementary data at *IJE* online).

In the sibling-sets sub-study, urban migrant siblings were more likely to be pre-hypertensive/hypertensive compared with rural siblings (aRR 2.01, 95% CI 1.30–3.09) but there was no evidence for increased risk by longer duration of stay (≥5 vs <5 years) among the urban sibling group (aRR 0.59, 95% CI 0.18–1.95; Table 5).

**Table 5.** Crude and adjusted risk ratios for cardiometabolic disorders within sibling sets

	Total Prevalence	Crude	Age- and sex-adjusted	Fully adjusted
	N(%)	IRR^a^	IRR^a^	IRR^a^^,^^b^
Rural siblings	231			
Urban siblings	129			
**Overweight and obesity^c^**				
Rural siblings	48(20.8)	1	1	1
Urban siblings	43(33.3)	1.47(0.94–2.29)	1.78(1.09–2.91)	2.06(1.03–4.12)
**Pre-hypertension**				
Rural siblings	78(33.8)	1	1	1
Urban siblings	82(63.7)	2.01(1.40–2.68)	2.08(1.45–2.99)	2.05(1.26–3.36)
**Hypertension**				
Rural siblings	11(4.8)	1	1	1
Urban siblings	12(9.3)	1.56 (0.64–3.81)	1.45 (0.52–4.02)	1.36(0.41–4.56)
**Hypertension and pre-hypertension^d^**				
Rural siblings	89(38.5)	1	1	1
Urban siblings	94(72.9)	1.93 (1.40–2.67)	1.99(1.43–2.75)	2.01(1.30–3.09)
**Duration of stay in urban location**				
**Overweight and obesity^e^**				
<5 years	31(40.8)	1	1	1
≥ 5 years	9(25.0)	2.88(0.98–8.50)	2.58(0.75–8.80)	3.11(0.68–14.16)
**Duration of stay in urban location**				
**Pre-hypertension^e^**				
<5 years	25(69.4)	1	1	1
≥5 years	49(25.0)	0.82(0.32–2.15)	0.61(0.22–1.71)	0.60(0.18–1.97)
**Duration of stay in urban location:**				
** Hypertension and pre-hypertension^f^**				
<5 years	28(77.8)	1	1	1
≥5 years	57(75.0)	0.86(0.33–2.19)	0.61(0.22–1.70)	0.59(0.18–1.95)

a IRR are risk ratios and 95% confidence intervals.

b Adjusted for age, sex, education, occupation, smoking, alcohol consumption and physical activity.

c Body mass index (BMI) according to World Health Organization (WHO) criteria. Underweight (BMI <18 kg/m^2^), normal (BMI 18–24.9 kg/m^2^), overweight (BMI 25–29.9 kg/m^2^), obese (BMI ≥30 kg/m^2^).

d Blood pressure (BP) according to WHO criteria. Hypertension is defined as systolic blood pressure ≥140 mm Hg, diastolic blood pressure ≥90 mm Hg or self-reported current anti-hypertensive medication use. Normal (BP < 130/80 mmHg), pre-hypertensive (BP = 131/81–139/89 mmHg), mild (BP = 140/90–159/99 mmHg), moderate (BP = 160/100–179/109 mmHg), severe (BP ≥ 180/110 mmHg).

e Relative risks obtained via logistic-regression analyses.

f Results for diabetes and multimorbidity have been omitted due to insufficient numbers of events.

### Blood glucose

Crude prevalence of diabetes in rural residents, rural-to-urban migrants and urban residents was 1.4, 2.9 and 1.9% for men and 1.5, 2.8 and 1.7% for women, respectively (Table 3). After WHO world-population age standardization, the prevalence of diabetes was 2.1, 5.6 and 5.3% in rural, rural-to-urban migrants and urban residents, respectively (Supplementary Table 6, available as Supplementary data at *IJE* online). Compared with urban residents, the risk of diabetes was substantively lower in rural residents (aRR men 0.44, 95% CI 0.29–0.68; aRR women 0.66, 95% CI 0.46–0.96) (Table 4) and equivalent in rural-to-urban migrants. Compared with the least educated and poor, those with most education and wealth experienced the highest diabetes risk in rural, urban and rural-to-urban migrants (Supplementary Tables 1–3, available as Supplementary data at *IJE* online). Although screening, diagnosis and medical treatment for diabetes were rare in participants at higher risk (age >40 years and overweight/obese), rural-to-urban migrants had greater access to screening, diagnosis and treatment for diabetes than either rural or urban residents (Supplementary Table 5, available as Supplementary data at *IJE* online).

In the sibling-set sub-study, statistical analysis of the association of migration with diabetes was not conducted due to the limited number of cases.

### Multimorbidity

Crude prevalence of multimorbidity in rural residents, rural-to-urban migrants and urban residents was 1.2, 3.7 and 1.8% for men and 3.1, 6.3 and 3.7% for women, respectively (Table 3). In all groups, the prevalence of multimorbidity increased with age before peaking at 50–59 years in women and 60–69 years in men (Supplementary Figure 2, available as Supplementary data at *IJE* online). In the total population, the multimorbidity risk was lower in rural residents (aRR 0.63, 95% CI 0.51–0.78) compared with urban residents. There was no evidence for risk differences between the urban residents and migrants (Table 4).

## Discussion

Our large population-level study in Malawi shows higher prevalences of overweight/obesity, hypertension and diabetes in rural-to-urban migrants than in either urban or rural residents. Consistent findings were observed in the sibling sub-study, with higher prevalences of CMD and precursor states in rural-to-urban migrant siblings compared with rural siblings. CMD risk was greater in urban than rural residents, comparable to findings elsewhere.^13^ Nonetheless, the observed higher risk of overweight/obesity and hypertension in rural-to-urban migrants compared with urban residents is novel.

Our population-level estimates are in line with national and regional prevalence estimates for urban and rural SSA.^7^^,^^24^^,^^25^ However, there are few published data on CMD in rural-to-urban migrant populations from LIC in SSA. Findings from several small SSA studies have shown inconsistent associations with risk for hypertension in rural residents compared with rural-to-urban migrants and comparisons with urban residents were not provided.^11^^,^^12^ In other African studies, urban residents have been shown to have higher risk of obesity, hypertension and diabetes compared with rural-to-urban migrants.^26^^,^^27^ Migration studies in MIC have also shown higher risk of CMD in urban residents compared with rural-to-urban migrants.^13^^,^^26^ In our study, the prevalence of overweight/obesity was high among all women, irrespective of migration status, corroborating recent findings on obesity in many SSA countries.^28^

CMD risk is largely attributed to modifiable risk factors.^4^^,^^13^ Previous migration studies have shown higher tobacco smoking, alcohol consumption, physical inactivity and psychosocial stress in urban residents compared with rural-to-urban migrants and rural residents.^13^^,^^26^ We also observed the highest tobacco and alcohol consumption in urban residents, largely in men (as consumption was rare in all women), but we did not observe material differences in physical activity between groups and the vast majority of the population met WHO physical-activity requirements. There was some variation in salt and sugar consumption, yet the highest prevalences were in rural residents. In contrast to findings from some MIC studies, variation in risk for CMD by migration status was not explained by modifiable risk factors in our study.^8–10^ Whereas we cannot exclude the potential effects of residual confounding, it is likely that a complex interplay of measured and unmeasured factors, including early-life exposures, environment, health care and psychosocial stress, contributes to the observed differences.

In our study, rural-to-urban migrants of higher socio-economic status and education experienced higher risk of CMD than those of lower socio-economic status, consistently with findings from other low and middle income countries and in stark contrast to the lower risk in higher socio-economic groups observed in developed countries.^9^^,^^29–31^ Using data from the whole study population, we found rural-to-urban migrants had wealth scores similar to those of urban residents, with a greater proportion in the higher-wealth categories, consistently with findings from MIC.^9^^,^^29^

We observed the highest burden of multimorbidity and access to screening, diagnosis and treatment for hypertension and diabetes among rural-to-urban migrants. It is unlikely that migration for health reasons drives our findings, as only 1% of participants reported migration for medical reasons, but the vast majority (43.4 and 35.6%) reported migration for work or study. To understand the reasons for different health-seeking patterns is beyond the scope of this study.

We utilized our detailed knowledge of migration patterns in adults and family linkages within the rural surveillance site to identify adult rural-to-urban migrant siblings. Our sibling-set study design minimized the effects of genetic, epigenetic and early-life-environment exposures that might have a bearing on CMD later in life.^32^ The higher risk of CMD and precursor states (overweight and pre-hypertension) in urban migrant siblings compared with rural siblings in our sibling cohort is comparable to findings from India.^9^ Most previous migrant studies have shown an increased CMD risk with longer duration of urban stay. Surprisingly, our sibling sub-study did not find associations between CMD risk and length of stay in urban areas ≥5 years. Nonetheless, our sibling study was small and findings should be interpreted with caution.

The large size of our population-level study and the matched sibling-set design of the sub-study, which limited the effects of unmeasured confounding, are notable strengths. Nonetheless, our study has several limitations. Available data are cross-sectional and rely on self-reported measures for place of birth and socio-demographic and lifestyle risk factors, hence our estimates may be affected by recall bias and reverse causation. Age-at-migration data were not available in the population-level study, hence we could not explore the extent to which CMD risk differ by duration of urban exposure. Further studies are needed to explore the extent to which contextual factors, including dietary patterns, epigenetics and adverse early-life conditions, influence CMD risk in rural-to-urban migrants in Malawi.

## Conclusion

In Malawi, rural-to-urban migration is associated with increased prevalence and risk of CMD compared with urban residency. For a country undergoing rapid urbanization and with limited resources to tackle CMDs, this poses a major public-health challenge. Development of prevention and management strategies that reach rural-to-urban migrants will be essential to delivering effective interventions for reducing and managing the burden of chronic disease in Malawi.

## Funding

This work was supported by the Wellcome Trust (098610/Z/12/Z).

## Supplementary Material

dyz198_Supplementary_Materials
