# Supplementary material for: Investigating associations between rural-to-urban migration and cardiometabolic disease in Malawi: a population-level study
Source: Int J Epidemiol. 2019 Oct 11;48(6):1850–62. doi: 10.1093/ije/dyz198 (PMC6929524; doi:10.1093/ije/dyz198)
Supplement: dyz198_Supplementary_Materials [file dyz198_supplementary_materials.docx]

Suppl.Figure 1: Flow chart of participation by site


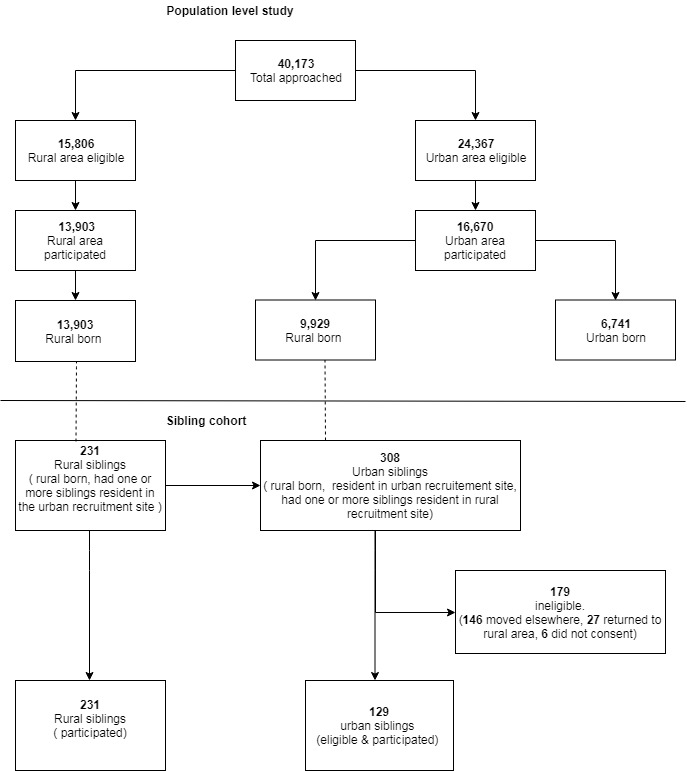


Suppl.Figure 2. Age-sex specific prevalence1 of a) hypertension b) overweight/obesity c) diabetes d) multimorbidity by migration status.


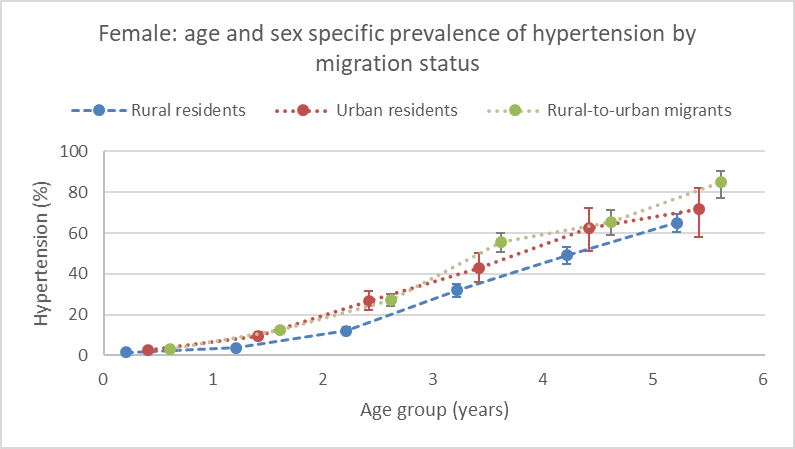

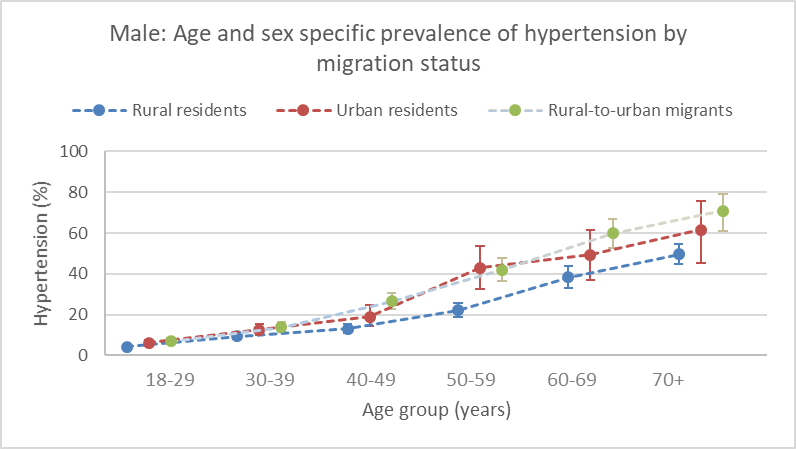


18-29 30-39 40-49 50-59 60-69 70+


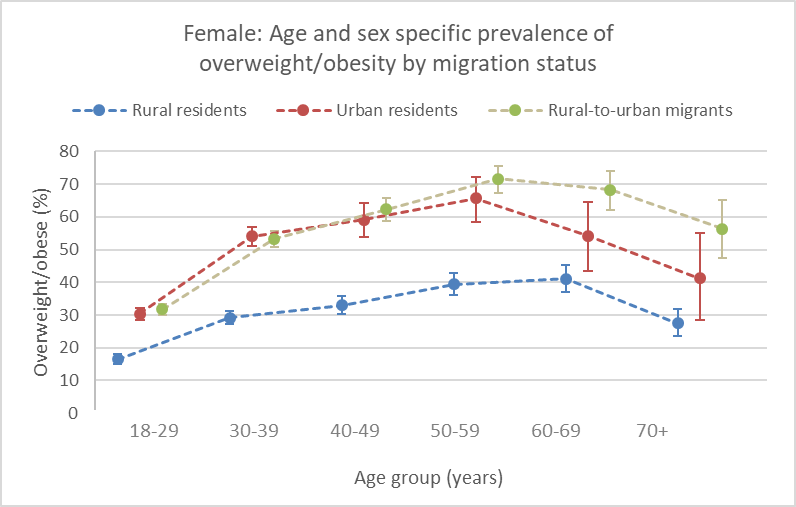

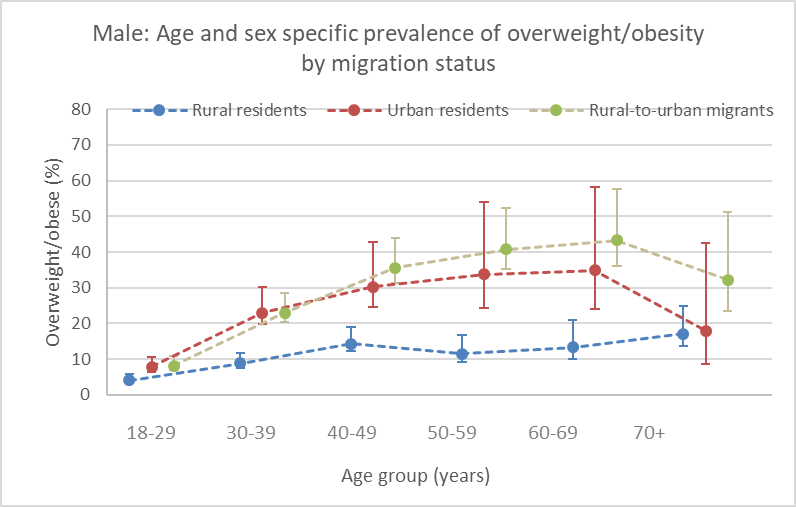

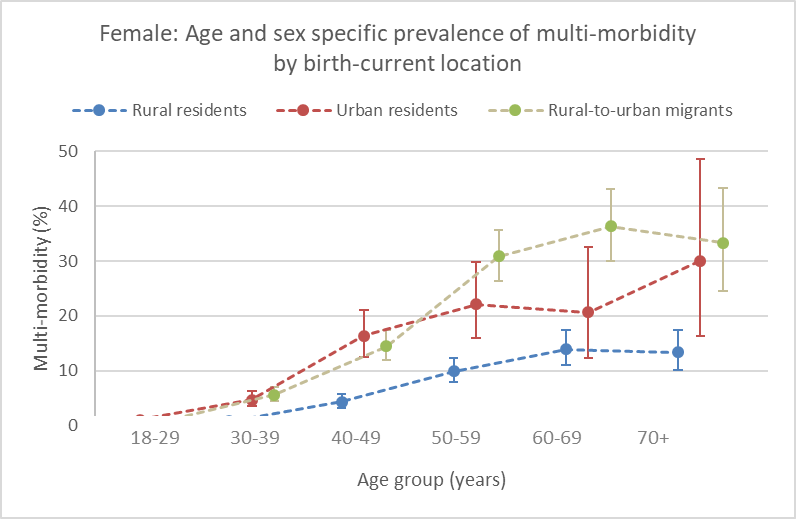

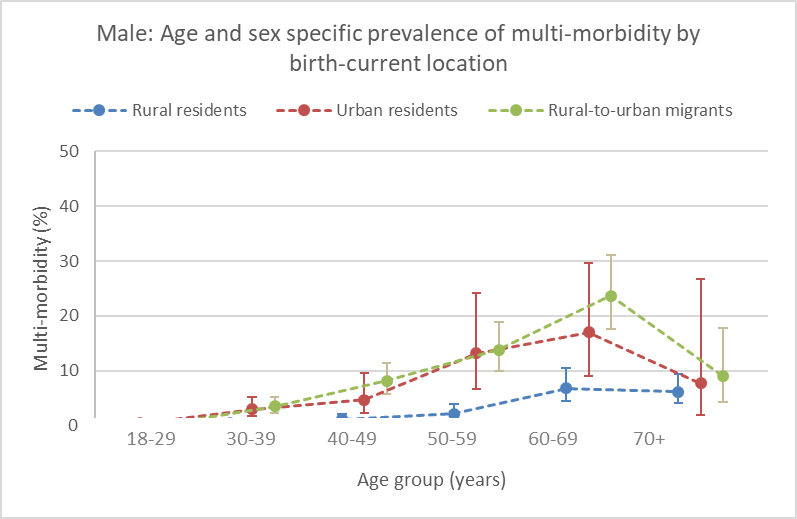

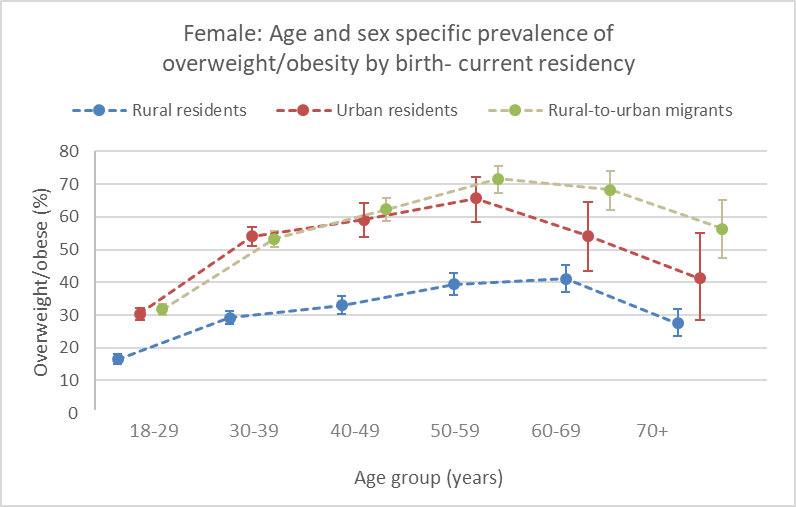

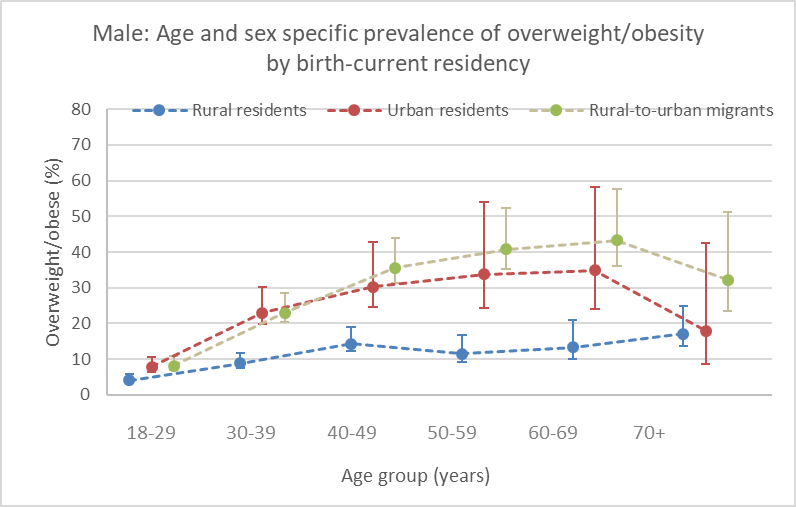

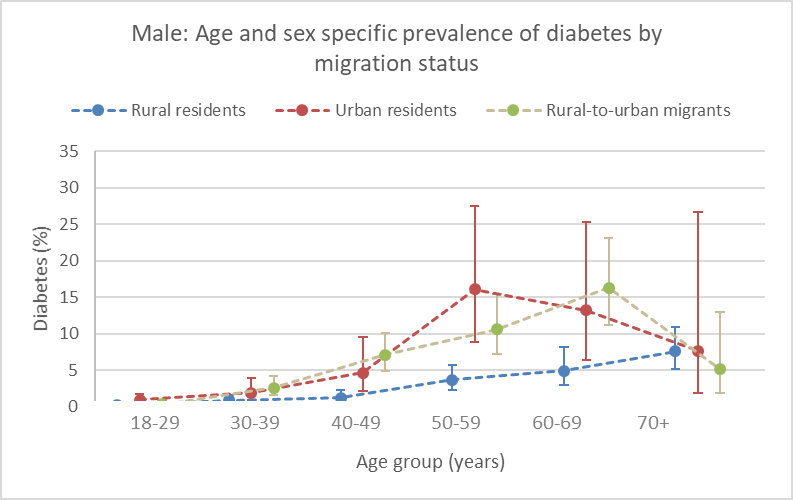

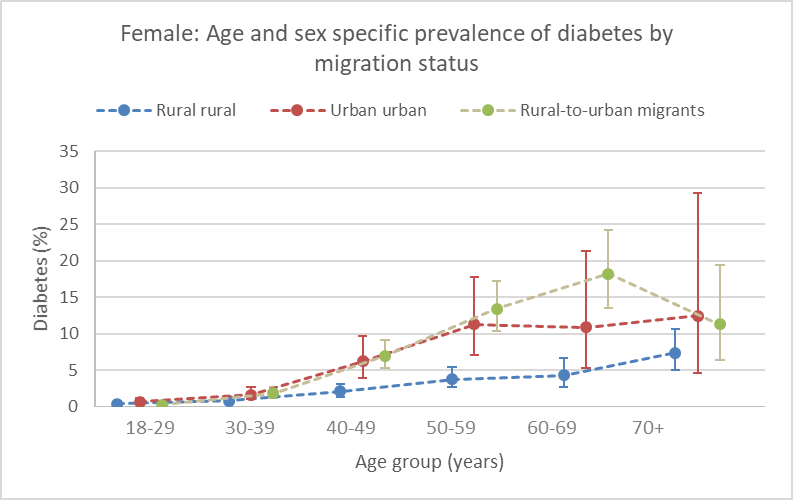

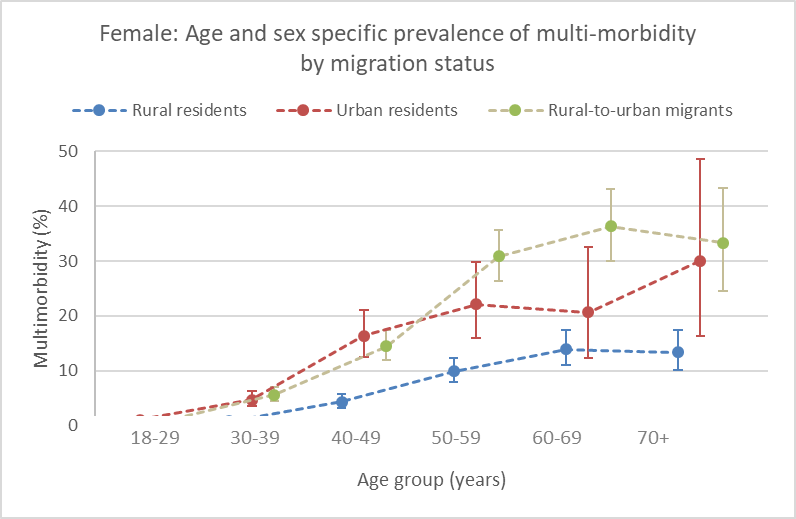

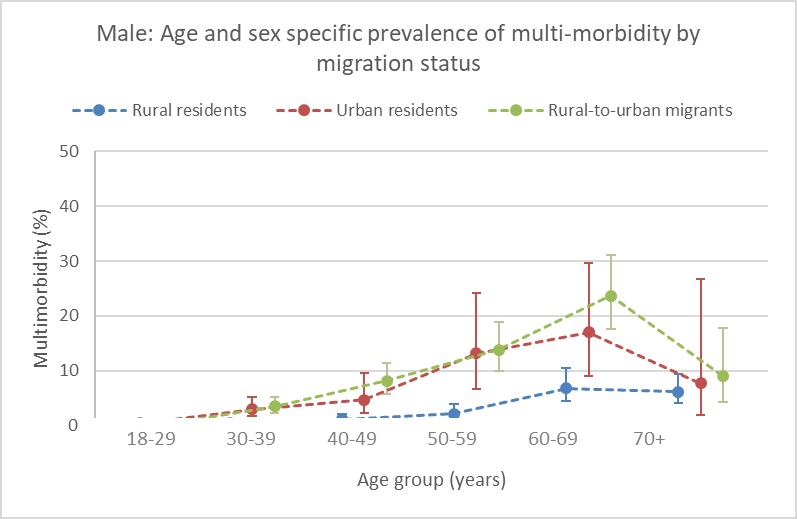


Suppl.Table 1. Association of education and wealth status with overweight, hypertension, diabetes and multimorbidity in rural-to-urban migrants.

|  | Total  N | Total  Age and sex adjusted^1^ | Total  Fully adjusted^1,2^ | Men  N | Men  Age-adjusted  RR^1^ | Men  Fully adjusted  RR^1,2^ | Women  N | Women  Age-adjusted  RR^1^ | Women  Fully adjusted  RR^1,2^ |
| --- | --- | --- | --- | --- | --- | --- | --- | --- | --- |
| **Rural-to-urban migrants** | 9929 |  |  | 3456 |  |  | 6473 |  |  |
| **Overweight/obese^3^** | | | | | | | | | |
| **Education**  None  Standard 1-5  Standard 6-8  Secondary  Tertiary  P value for trend  **Wealth quintile**  Poorest  2  3  4  Wealthiest  P value for trend | 149(4.2%)  363(10.2%)  842(23.7%)  1548 (43.5%)  661(18.6%)  332(9.3%)  416(11.7%)  576(16.2%)  1014(28.5%)  1225(34.4%) | 1  1.29(1.10-1.51)  1.41(1.22-1.62)  1.53(1.32-1.76)  2.16(1.87-2.51)  <0.001  1  1.14(1.11-1.28)  1.34(1.20-1.51)  1.56(1.40-1.71)  1.67(1.51-1.84)  <0.001 | 1  1.21(1.05-1.40)  1.26(1.09-1.44)  1.33(1.17-1.53)  1.78(1.54-2.07)  <0.001  1  1.12(0.99-1.26)  1.32(1.18-1.48)  1.48(1.33-1.64)  1.59(1.44-1.77)  <0.001 | 4(0.6%)  22(3.1%)  89(12.3%)  350(48.5%)  257(35.6%)  35(4.9%)  56(7.8%)  111(15.4%)  213(29.5%)  307(42.5%) | 1  2.10(0.81-5.50)  3.68(1.52-8.97)  5.29(2.21-12.71)  9.32(3.88-22.34)  <0.001  1  1.17(0.78-1.76)  1.80(1.25-2.60)  2.38(1.69-3.36)  2.93(2.09-4.08)  <0.001 | 1  1.70(0.70-4.12)  2.56(1.14-5.79)  3.43(1.54-7.66)  5.11(2.28-11.44)  <0.001  1  1.21(0.81-1.79)  1.73(1.22-2.47)  2.09(1.49-2.93)  2.61(1.87-3.63)  <0.001 | 145(5.1%)  341(12.0%)  753(26.5%)  1198(42.2%)  404(14.2%)  297(10.6%)  360(12.7%)  465(16.4%)  801(28.2%)  918(32.3%) | 1  1.25(1.08-1.46)  1.30(1.14-1.49)  1.34(1.16-1.54)  1.64(1.41-1.89)  <0.001  1  1.15(1.02-1.30)  1.30(1.16-1.46)  1.46(1.31-1.62)  1.48(1.33-1.64)  <0.001 | 1  1.19(1.03-1.38)  1.18(1.03-1.35)  1.18(1.03-1.36)  1.42(1.23-1.66)  <0.001  1  1.14(1.00-1.29)  1.16(1.15-1.46)  1.44(1.29-1.61)  1.49(1.34-1.66)  <0.001 |
| **Hypertension^4^** | | | | | | | | | |
| **Education**  None  Standard 1-5  Standard 6-8  Secondary  Tertiary  P value for trend  **Wealth quintile**  Poorest  2  3  4  Wealthiest  P value for trend | 117(6.9%)  212(12.7%)  405(24.2%)  645(38.6%)  294(17.6%)  127(7.6%)  193(11.5%)  263(15.7%)  499(29.8%)  591(35.3%) | 1  1.16(0.95-1.43)  1.26(1.05-1.52)  1.34(1.11-1.62)  1.58(1.29-1.93)  <0.001  1  1.30(1.05-1.61)  1.41(1.16-1.72)  1.65(1.37-1.98)  1.79(1.49-2.14)  <0.001 | 1  1.09(0.89-1.33)  1.13(0.94-1.36)  1.18(0.97-1.43)  1.29(1.05-1.61)  0.014  1  1.28(1.04-1.59)  1.38(1.13-1.69)  1.62(1.35-1.96)  1.75(1.45-2.11)  <0.001 | 13(2.1%)  33(5.1%)  118(18.2%)  312(48.2%)  172(26.5%)  52(8.0%)  78(12.0%)  105(16.2%)  177(27.3%)  236(36.4%) | 1  0.65(0.37-1.13)  0.75(0.74-1.22)  0.55(0.34-0.88)  0.82(0.51-1.32)  <0.001  1  1.06(0.76-1.48)  1.09(0.79-1.51)  1.25(0.94-1.68)  1.46(1.10-1.92)  0.003 | 1  1.13(0.76-1.68)  1.19(0.83-1.69)  1.46(0.99-2.15)  1.24(0.78-1.98)  <0.001  1  1.07(0.77-1.48)  1.04(0.75-1.43)  1.13(0.84-1.52)  1.29(0.97-1.73)  0.087 | 104(10.2%)  179(17.5%)  287(28.0%)  333(32.5%)  122(11.9%)  75(7.3%)  115(11.2%)  158(15.4%)  322(31.4%)  355(34.6%) | 1  0.76(0.62-0.93)  0.52(0.43-0.62)  0.33(0.27-0.39)  0.48(0.28-0.59)  <0.001  1  1.48(1.13-1.94)  1.64(1.27-2.12)  1.91(1.51-2.43)  1.99(1.58-2.52)  <0.001 | 1  1.18(0.95-1.48)  1.13(0.92-1.41)  1.18(0.94-1.48)  1.23(0.94-1.62)  0.179  1  1.45(1.11-1.90)  1.58(1.23-2.04)  1.90(1.49-2.42)  1.98(1.56-2.53)  <0.001 |
| **Diabetes^5^** | | | | | | | | | |
| **Education**  None  Standard 1-5  Standard 6-8  Secondary  Tertiary  P value for trend  **Wealth quintile**  Poorest  2  3  4  Wealthiest  P value for trend | 12(4.3%)  26(9.3%)  67(23.8%)  102(36.3%)  74(26.3%)  7(2.5%)  16(5.7%)  42(14.9%)  89(31.7%)  127(45.2%) | 1  4.64(0.54-40.16)  10.01(1.26-79.46)  19.32(2.43-153.88)  40.04(5.01-319.90)  <0.001  1  1.82(0.75-4.46)  3.75(1.68-8.34)  4.68(2.17-10.09)  6.23(2.92-13.33)  <0.001 | 1  1.22(0.58-2.56)  1.79(0.92-3.51)  1.86(0.93-3.69)  2.96(1.46-5.97)  0.001  1  1.70(0.69-4.17)  3.29(1.47-7.39)  3.74(1.71-8.23)  4.83(2.22-10.52)  0.001 | 0.00  4(3.9%)  14(13.9%)  44(43.6%)  39(38.6%)  3(2.9%)  3(2.9%)  9(8.9%)  38(37.6%)  48(47.5%) | 1  1  0.97(0.17-5.64)  5.53(1.59-19.16)  13.33(3.92-45.36)  <0.001  1  0.59(0.12-2.99)  1.41(0.38-5.36)  3.95(1.19-13.11)  4.35(1.33-14.25)  <0.001 | 1  1  1.25(0.37-4.27)  1.49(0.44-4.99)  2.22(0.66-7.56)  0.183  1  0.56(0.11-2.85)  1.21(0.51-33.37)  3.09(0.89-10.63)  3.34(0.99-11.22)  0.004 | 12(6.7%)  22(12.2%)  53(29.4%)  58(32.2%)  35(19.4%)  4(2.2%)  13(7.2%)  33(18.3%)  51(28.3%)  79(43.9%) | 1  4.98(0.51-48.67)  11.31(1.38-92.65)  17.84(2.11-150.77)  31.31(3.62-270.71)  <0.001  1  3.06(1.01-9.31)  5.94(2.13-16.54)  4.97(1.81-13.61)  7.55(2.79-20.39)  <0.001 | 1  1.15(0.53-2.47)  1.80(0.90-3.59)  1.78(0.86-3.69)  2.89(1.32-6.33)  0.004  1  2.79(0.91-8.57)  5.21(1.84-14.72)  3.93 (1.39-11.07)  5.81(2.09-16.15)  <0.001 |
| **Multimorbidity^6^** | | | | | | | | | |
| **Education**  None  Standard 1-5  Standard 6-8  Secondary  Tertiary  P value for trend  **Wealth quintile**  Poorest  2  3  4  Wealthiest  P value for trend | 26(4.8%)  66(12.3%)  138(25.7%)  183(34.1%)  124(23.1%)  16(2.5%)  29(5.7%)  75(14.9%)  169(31.7%)  248(46.2%) | 1  1.73(1.06-2.82)  2.31(1.47-3.66)  2.63(1.67-4.17)  4.69(2.96-7.44)  <0.001  1  1.46(0.78-2.71)  2.87(1.66-4.96)  3.79(2.25-6.39)  5.42(3.23-9.09)  <0.001 | 1  1.45(0.88-2.37)  1.72(1.04-2.74)  1.89(1.12-3.04)  3.11(1.47-5.07)  <0.001  1  1.35(0.73-1.62)  2.57(1.49-4.45)  3.18(1.87-5.41)  4.39(2.60-7.44)  <0.001 | 0.00  2(1.6%)  15(11.6%)  54(41.9%)  58(44.9%)  5(3.9%)  4(3.1%)  10(7.8%)  39(30.2%)  71(55.1%) | -  1  4.16(0.84-20.59)  7.54(1.57-36.28)  17.04(3.58-81.16)  <0.001  1  0.45(0.11-1.73)  0.91(0.29-2.82)  2.31(0.87-6.13)  3.84(1.47-9.96)  <0.001 | -  1  2.87(0.59-13.96)  4.87(1.05-23.58)  8.86(1.84-42.67)  0.004  1  0.42(0.11-1.56)  0.69(0.22-2.15)  1.36(0.49-3.73)  2.11(0.79-5.66)  0.022 | 26(6.4%)  64(15.7%)  123(30.2%)  129(31.6%)  66(16.2%)  11(2.7%)  25(6.2%)  65(15.9%)  130(31.9%)  177(43.4%) | 1  1.79(1.09-2.95)  2.27(1.43-3.63)  2.38(1.47-3.85)  3.45(2.11-5.67)  <0.001  1  2.05(1.00-4.19)  3.97(2.09-7.55)  4.46(2.39-8.31)  6.03(3.26-11.17)  <0.001 | 1  1.47(0.89-2.39)  1.67(1.04-2.66)  1.68(1.04-2.73)  2.39(1.41-4.03)  0.001  1  1.91(0.94-3.89)  3.61(1.90-6.84)  3.97(2.12-7.45)  5.26(2.82-9.81)  <0.001 |

^1^ RR are risk ratios (95% Confidence Intervals).

^2^ Adjusted for age, sex, education (none, standard 1-5, standard 6-8, secondary, tertiary), occupation (not working, housework, farming/fishing, self-employed, employed) and wealth status (poorest, 2, 3, 4, wealthiest), as appropriate.

^3^ Overweight defined as BMI 25-29.9 kg/m^2^ and obesity defined as BMI≥30 kg/m^2^.

^4^ Hypertension is defined as SBP ≥140 mmHg, DBP ≥90 mmHg, or self-reported that currently using antihypertensive medication.

^5^ Diabetes mellitus (DM) defined as FBG ≥7.0 mmol/L, or current use of medication prescribed to treat diabetes mellitus or self-reported.

^6^ Multimorbidity defined as the presence of two or more of hypertension, diabetes, and obesity

Suppl.Table 2. Association of education and wealth status with overweight, hypertension, diabetes and multimorbidity in urban residents.

|  | Total  N | Total  Age and sex adjusted^1^ | Total  Fully adjusted^1^ | Men  N (%) | Men  Age-adjusted  RR^1^ | Men  Fully adjusted  RR^1^ | Women  N (%) | Women  Age-adjusted  RR^1^ | Women  Fully adjusted  RR^1,2^ |
| --- | --- | --- | --- | --- | --- | --- | --- | --- | --- |
| **Urban residents** | 6741 |  |  | 2348 |  |  | 4393 |  |  |
| **Overweight/obese^3^** | | | | | | | | | |
| **Education**  None  Standard 1-5  Standard 6-8  Secondary  Tertiary  P value for trend  **Wealth quintile**  Poorest  2  3  4  Wealthiest  P value for trend | 80(3.8%)  147(7.1%)  367(17.6%)  1060(50.9%)  430(20.6%)  232(11.1%)  249(11.9%)  330(15.8%)  557(26.7%)  716(34.4%) | 1  1.22(0.95-1.56)  1.47(1.18-1.83)  1.71(1.38-2.13)  2.17(1.73-2.71)  <0.001  1  1.15(0.99-1.35)  1.31(1.14-1.51)  1.54(1.35-1.75)  1.79(1.58-2.02)  <0.001 | 1  1.13(0.89-1.42)  1.26(1.02-1.56)  1.38(1.12-1.72)  1.59(1.27-2.01)  <0.001  1  1.09(0.94-1.28)  1.23(1.07-1.43)  1.42(1.24-1.63)  1.66(1.46-1.90)  <0.001 | 6(1.7%)  11(3.1%)  32(8.9%)  165(46.2%)  143(40.1%)  27(7.6%)  27(7.6%)  44(12.3%)  107(29.9%)  152(42.6%) | 1  0.83(0.29-2.38)  1.27(0.49-3.28)  2.19(0.86-5.59)  4.01(1.58-10.18)  <0.001  1  1.08(0.64-1.80)  1.31(0.83-2.08)  2.23(1.48-3.34)  2.74(1.85-4.06)  <0.001 | 1  0.75(0.28-2.01)  0.95(0.39-2.32)  1.45(0.61-3.45)  2.22(0.93-5.32)  0.021  1  0.94(0.57-1.56)  1.16(0.74-1.81)  1.77(1.18-2.65)  2.17(1.46-3.22)  <0.001 | 74(4.3%)  136(7.9%)  335(19.4%)  895(51.8%)  287(16.6%)  205(11.9%)  222(12.9%)  286(16.6%)  450(26.1%)  564(32.7%) | 1  1.24(0.98-1.58)  1.46(1.18-1.82)  1.63(1.32-2.01)  1.73(1.39-2.17)  <0.001  1  1.18(1.01-1.38)  1.33(1.15-1.54)  1.44(1.26-1.65)  1.64(1.44-1.87)  <0.001 | 1  1.15(0.91-1.45)  1.28(1.03-1.58)  1.34(1.08-1.65)  1.31(1.04-1.65)  0.008  1  1.13(0.96-1.33)  1.28(1.10-1.49)  1.39(1.21-1.59)  1.62(1.41-1.86)  <0.001 |
| **Hypertension^4^** | | | | | | | | | |
| **Education**  None  Standard 1-5  Standard 6-8  Secondary  Tertiary  P value for trend  **Wealth quintile**  Poorest  2  3  4  Wealthiest  P value for trend | 53(7.2%)  68(9.3%)  156(21.3%)  308(42.1%)  148(20.2%)  86(11.7%)  86(11.7%)  130(17.7%)  192(26.2%)  239(32.6%) | 1  0.97(0.85-1.11)  1.05(0.91-1.22)  1.11(0.94-1.32)  1.81(1.38-2.39)  <0.001  1  1.13(0.86-1.51)  1.59(1.23-2.06)  1.67(1.31-2.12)  1.83(1.45-2.31)  <0.001 | 1  0.95(0.67-1.35)  1.23(0.91-1.67)  1.37(0.99-1.89)  1.56(1.09-2.23)  0.003  1  1.07(0.81-1.42)  1.46(1.13-1.89)  1.48(1.16-1.90)  1.62(1.26-2.07)  <0.001 | 6(2.1%)  12(4.2%)  51(17.8%)  126(44.1%)  91(31.8%)  30(10.5%)  29(10.1%)  45(15.7%)  79(27.6%)  103(36.1%) | 1  0.68(0.28-1.59)  1.04(0.49-2.24)  0.51(0.24-1.06)  0.87(0.41-1.83)  0.467  1  1.08(0.66-1.77)  1.28(0.83-2.00)  1.63(1.09-2.44)  1.79(1.21-2.63)  <0.001 | 1  0.92(0.39-2.19)  2.42(1.12-5.23)  2.34(1.07-5.09)  3.25(1.47-7.21)  <0.001  1  0.95(0.58-1.56)  1.15(0.74-1.79)  1.32(0.88-1.99)  1.41(0.94-2.11)  0.035 | 47(10.5%)  56(12.5%)  105(23.5%)  182(40.7%)  57(12.8%)  56(12.5%)  57(12.8%)  85(19.0%)  113(25.3%)  136(30.4%) | 1  0.66(0.46-0.93)  0.53(0.39-0.72)  0.32(0.24-0.43)  0.34(0.24-0.49)  <0.001  1  1.15(0.82-1.64)  1.81(1.32-2.49)  1.65(1.22-2.23)  1.84(1.38-2.45)  <0.001 | 1  1.13(0.76-1.68)  1.18(0.83-1.69)  1.46(0.99-2.15)  1.24(0.78-1.98)  0.207  1  1.03(0.73-1.47)  1.61(1.17-2.22)  1.43(1.04-1.97)  1.59(1.17-2.18)  <0.001 |
| **Diabetes^5^** | | | | | | | | | |
| **Education**  None  Standard 1-5  Standard 6-8  Secondary  Tertiary  P value for trend  **Wealth quintile**  Poorest  2  3  4  Wealthiest  P value for trend | 1(0.8%)  6(5.0%)  21(17.5%)  52(43.3%)  40(33.3%)  6(5.0%)  6(5.0%)  9(7.5%)  41(34.2%)  58(48.3%) | 1  4.64(0.54-40.16)  10.01(1.26-79.46)  19.33(2.43-153.88)  40.03(5.01-319.90)  <0.001  1  1.01(0.34-3.07)  1.19(0.42-3.37)  4.31(1.81-10.25)  5.65(2.42-13.24)  <0.001 | 1  4.74(0.57-39.56)  7.71(0.98-59.57)  11.21(1.49-84.54)  17.06(2.24-130.04)  0.002  1  0.75(0.24-2.37)  0.89(0.32-2.49)  2.51(1.04-6.03)  2.92(1.23-6.94)  <0.001 | 0.00  2(4.6%)  2(4.6%)  18(40.9%)  22(50.1%)  1(2.3%)  1(2.3%)  6(13.6%)  16(36.4%)  20(45.5%) | -  1  0.97(0.17-5.64)  5.52(1.59-19.16)  13.33(3.92-45.36)  <0.001  1  1.07(0.08-15.03)  4.40(0.53-36.03)  10.97(1.49-80.88)  11.03(1.51-80.11)  <0.001 | -  1  0.74(0.13-4.32)  3.01(0.75-12.22)  5.75(1.42-23.31)  <0.001  1  0.85(0.06-11.01)  3.25(0.46-23.03)  5.40(0.81-36.19)  4.70(0.68-32.48)  0.022 | 1(1.3%)  4(5.3%)  19(29.4%)  34(44.7%)  18(23.7%)  5(6.6%)  5(6.6%)  3(3.9%)  25(32.9%)  38(50.0%) | 1  4.98(0.51-48.68)  11.31(1.38-92.65)  17.84(2.11-150.77)  31.31(3.62-270.72)  <0.001  1  0.99(0.29-3.34)  0.52(0.12-2.21)  3.01(1.14-7.93)  4.63(1.80-11.89)  <0.001 | 1  4.49(0.48-42.01)  8.92(1.13-70.33)  10.05(1.25-80.56)  11.59(1.38-97.69)  0.013  1  0.67(0.18-2.41)  0.37(0.09-1.52)  1.73(0.64-4.64)  2.45(0.94-6.42)  0.010 |
| **Multimorbidity^6^** | | | | | | | | | |
| **Education**  None  Standard 1-5  Standard 6-8  Secondary  Tertiary  P value for trend  **Wealth quintile**  Poorest  2  3  4  Wealthiest  P value for trend | 5(2.4%)  21(10.2%)  35(17.1%)  89(43.4%)  55(26.8%)  5(2.4%)  9(4.4%)  28(13.7%)  69(33.7%)  94(45.9%) | 1  0.94(0.62-1.41)  1.56(1.03-2.37)  2.27(1.41-3.66)  4.84(2.38-9.83)  <0.001  1  1.56(1.09-2.25)  1.79(1.25-2.57)  3.09(2.20-4.34)  3.63(2.46-5.36)  <0.001 | 1  4.37(1.59-12.07)  2.63(0.97-7.14)  4.42(1.59-12.22)  5.96(2.09-16.99)  0.002  1  1.39(0.46-4.28)  3.86(1.45-10.27)  6.65(2.56-17.33)  7.95(3.06-20.64)  <0.001 | 0.00  2(4.8%)  2(4.8%)  15(35.7%)  23(54.8%)  1(2.4%)  1(2.4%)  3(7.2%)  17(40.5%)  20(47.6%) | -  1  1.15(0.19-6.69)  6.54(1.84-23.19)  20.47(6.19-67.59)  <0.001  1  1.08(0.07-15.06)  2.06(0.22-19.31)  14.03(1.84-106.68)  12.55(1.68-93.71)  <0.001 | -  1  0.58(0.99-3.48)  2.34(0.55-9.99)  5.79(1.35-24.89)  0.010  1  0.74(0.05-10.24)  1.71(0.22-12.95)  7.23(1.07-48.96)  5.49(0.78-38.44)  0.009 | 5(6.4%)  19(11.7%)  33(20.3%)  74(45.4%)  32(19.6%)  4(2.5%)  8(4.9%)  25(15.3%)  52(31.9%)  74(45.4%) | 1  5.98(2.11-16.92)  4.38(1.64-11.76)  9.03(3.30-24.66)  12.86(4.59-36.03)  <0.001  1  1.99(0.59-6.66)  5.78(1.94-17.27)  8.12(2.84-23.20)  11.93(4.20-33.86)  <0.001 | 1  4.86(1.68-14.01)  2.79(0.99-7.83)  4.21(1.45-12.21)  3.85(1.24-11.89)  0.004  1  1.47(0.42-5.13)  4.36(1.46-13.05)  5.97(2.04-17.51)  8.22(2.82-24.01)  <0.001 |

^1^ RR are risk ratios (95% Confidence Intervals)

^2^ Adjusted for age, sex, education (none, standard 1-5, standard 6-8, secondary, tertiary), occupation (not working, housework, farming/fishing, self-employed, employed) and wealth status (poorest, 2, 3, 4, wealthiest), as appropriate

^3^ Overweight defined as BMI 25-29.9 kg/m^2^ and obesity defined as BMI≥30 kg/m^2^

^4^ Hypertension is defined as SBP ≥140 mmHg, DBP ≥90 mmHg, or self-reported that currently using antihypertensive medication.

^5^ Diabetes mellitus (DM) defined as FBG ≥7.0 mmol/L, or current use of medication prescribed to treat diabetes mellitus or self-reported.

^6^ Multimorbidity defined as the presence of two or more of hypertension, diabetes, and obesity.

Suppl.Table 3. Association of education and wealth status with overweight, hypertension, diabetes and multimorbidity in rural residents.

|  | Total  N | Total  Age and sex adjusted^1^ | Total  Fully adjusted^1,2^ | Men  N | Men  Age-adjusted  RR^1^ | Men  Fully adjusted  RR^1,2^ | Women  N | Women  Age-adjusted  RR^1^ | Women  Fully adjusted  RR^1,2^ |
| --- | --- | --- | --- | --- | --- | --- | --- | --- | --- |
| **Rural residents** | 13,903 |  |  | 5,864 |  |  | 8,039 |  |  |
| **Overweight/obsese^3^** | | | | | | | | | |
| **Education**  None  Standard 1-5  Standard 6-8  Secondary  Tertiary  P value for trend  **Wealth quintile**  Poorest  2  3  4  Wealthiest  P value for trend | 150(5.8%)  415(16.2%)  1220(47.6%)  722 (28.2%)  55(2.15%)  548(21.4%)  670(26.2%)  574(22.4%)  435(16.9%)  335(13.1%) | 1  1.18(1.01-1.39)  1.58(1.35-1.86)  1.90(1.59-2.26)  2.61(1.97-3.44)  <0.001  1  1.21(1.09-1.34)  1.40(1.27-1.56)  1.53(1.53-1.71)  2.21(1.97-2.48)  <0.001 | 1  1.13(0.95-1.34)  1.39(1.18-1.63)  1.48(1.24-1.77)  1.27(0.94-1.72)  0.021  1  1.15(1.04-1.27)  1.33(1.20-1.48)  1.41(1.27-1.58)  1.97(1.74-2.22)  <0.001 | 8(1.5%)  48(9.2%)  187(35.8%)  250(47.9%)  29(5.6%)  69(13.2%)  134(25.7%)  133(25.5%)  85(16.3%)  101(19.4%) | 1  1.18(0.59-2.39)  1.80(0.92-3.52)  2.67(1.37-5.22)  3.67(1.76-7.68)  <0.001  1  1.49(1.12-1.97)  1.88(1.42-2.49)  1.81(1.34-2.46)  3.84(2.88-5.11)  <0.001 | 1  1.08(0.53-2.19)  1.48(0.76-2.88)  1.80(0.92-3.54)  1.49(0.71-3.16)  0.099  1  1.33(1.01-1.76)  1.67(1.26-2.22)  1.49(1.10-2.04)  3.01(2.23-4.04)  <0.001 | 142(6.9%)  367(17.9%)  1033(50.6%)  472(23.1%)  26(1.3%)  479(23.5%)  536(26.3%)  441(21.6%)  350(17.2%)  234(11.5%) | 1  1.16(0.98-1.38)  1.49(1.27-1.76)  1.63(1.35-1.96)  2.04(1.47-2.85)  <0.001  1  1.15(1.04-1.29)  1.30(1.17-1.46)  1.47(1.32-1.66)  1.83(1.61-2.07)  <0.001 | 1  1.12(0.94-1.33)  1.33(1.13-1.58)  1.35(1.12-1.63)  1.16(0.81-1.67)  0.199  1  1.12(1.01-1.24)  1.26(1.13-1.41)  1.41(1.26-1.58)  1.70(1.49-1.94)  <0.001 |
| **Hypertension^4^** | | | | | | | | | |
| **Education**  None  Standard 1-5  Standard 6-8  Secondary  Tertiary  P value for trend  **Wealth quintile**  Poorest  2  3  4  Wealthiest  P value for trend | 227(12.0%)  490(25.9%)  734(38.9%)  385(20.4%)  52(2.8%)  488(25.9%)  497(26.3%)  406(21.5%)  323(17.1%)  174(9.22%) | 1  0.97(0.84-1.11)  1.06(0.91-1.22)  1.11(0.94-1.32)  1.82(1.37-2.39)  <0.001  1  1.07(0.97-1.19)  1.08(0.95-1.22)  1.28(1.12-1.45)  1.30(1.11-1.52)  <0.001 | 1  0.96(0.84-1.11)  1.01(0.87-1.17)  1.03(0.86-1.23)  1.48(1.11-1.98)  0.008  1  1.05(0.94-1.18)  1.06(0.94-1.19)  1.23(1.08-1.41)  1.23(1.05-1.43)  0.001 | 25(3.2%)  135(17.2%)  312(39.6%)  276(35.1%)  39(4.9%)  161(20.5%)  212(26.9%)  185(23.5%)  149(18.9%)  80(10.2%) | 1  0.84(0.58-1.22)  0.85(0.36-0.73)  0.43(0.30-0.62)  0.88(0.57-1.37)  0.045  1  1.00(0.83-1.21)  1.05(0.87-1.27)  1.28(1.05-1.56)  1.25(0.99-1.59)  0.008 | 1  1.10(0.74-1.64)  1.22(0.83-1.81)  1.29(0.87-1.94)  1.69(1.06-2.72)  0.017  1  0.97(0.80-1.17)  1.01(0.83-1.23)  1.19(0.97-1.46)  1.15(0.89-1.46)  0.077 | 202(18.4%)  355(32.2%)  422(38.3%)  109(9.9%)  13(1.2%)  327(29.7%)  285(25.9%)  221(20.1%)  174(15.8%)  94(8.5%) | 1  0.63(0.55-0.73)  0.25(0.22-0.29)  0.13(0.11-0.16)  0.41(0.24-0.68)  <0.001  1  1.19(1.03-1.37)  1.19(1.02-1.38)  1.36(1.16-1.61)  1.43(1.16-1.76)  <0.001 | 1  1.05(0.95-1.48)  1.21(0.92-1.41)  1.11(0.94-1.48)  2.51(0.94-1.62)  0.003  1  1.16(1.00-1.34)  1.16(0.99-1.35)  1.32(1.12-1.56)  1.33(1.08-1.63)  0.002 |
| **Diabetes^5^** | | | | | | | | | |
| **Education**  None  Standard 1-5  Standard 6-8  Secondary  Tertiary  P value for trend  **Wealth quintile**  Poorest  2  3  4  Wealthiest  P value for trend | 12(5.8%)  39(18.8%)  92(44.2%)  59(28.4%)  6(2.9%)  39(18.8%)  53(25.5%)  44(21.2%)  40(19.2%)  32(15.4%) | 1  1.44(0.76-2.76)  2.57(1.37-4.81)  3.60(1.80-7.22)  4.61(1.67-12.71)  <0.001  1  1.32(0.87-1.99)  1.36(0.88-2.09)  1.83(1.17-2.83)  2.68(1.69-4.24)  <0.001 | 1  1.40(0.74-2.66)  2.32(1.25-4.29)  3.03(1.52-6.01)  3.35(1.11-10.12)  0.008  1  1.22(0.81-1.85)  1.24(0.80-1.91)  1.56(1.02-2.39)  2.18(1.38-3.45)  0.001 | 11(13.1%) 20(23.8%)  21(25.0%)  18(21.4%)  14(16.7%)  1(1.2%) 9(10.7%)  34(40.5%)  36(42.8%)  4(4.8%) | 1  1.91(0.23-15.49)  4.72(0.62-35.65)  6.64(0.86-51.06)  6.79(0.74-62.44)  0.029  1  1.29(0.61-2.69)  1.54(0.74-3.21)  2.04(0.97-4.27)  2.77(1.27-6.04)  0.003 | 1  1.86(0.23-15.18)  4.34(0.58-32.68)  5.98(0.78-45.55)  5.41(0.58-50.47)  0.051  1  1.18(0.57-2.45)  1.33(0.64-2.75)  1.66(0.82-3.34)  2.35(1.09-5.04)  0.014 | 28(22.6%)  33(26.61%)  23(18.6%)  22(17.7%)  18(14.5%)  11(8.9%)  30(24.2%)  58(46.8%)  23(18.6%)  2(1.6%) | 1  1.47(0.74-2.93)  2.28(1.14-4.60)  3.04(1.29-7.13)  6.32(1.37-29.11)  0.007  1  1.35(0.82-2.24)  1.24(0.72-2.15)  1.71(0.98-2.97)  2.65(1.49-4.74)  <0.001 | 1  1.47(0.75-2.92)  2.12(1.05-4.27)  2.49(1.06-5.88)  5.59(0.47-66.71)  0.128  1  1.27(0.77-2.09)  1.18(0.68-2.04)  1.52(0.87-2.62)  2.19(1.23-3.92)  0.007 |
| **Multimorbidity^6^** | | | | | | | | | |
| **Education**  None  Standard 1-5  Standard 6-8  Secondary  Tertiary  P value for trend  **Wealth quintile**  Poorest  2  3  4  Wealthiest  P value for trend | 38(12.1%)  72(22.9%)  129(41.1%)  65(20.7%)  10(3.2%)  52(16.7%)  72(22.9%)  65(20.7%)  78(24.8%)  47(14.9%) | 1  0.94(0.62-1.41)  1.56(1.02-2.37)  2.27(1.41-3.66)  4.84(2.38-9.83)  <0.001  1  1.56(1.09-2.23)  1.79(1.25-2.57)  3.09(2.20-4.34)  3.63(2.46-5.36)  <0.001 | 1  0.89(0.58-1.35)  1.29(0.85-1.98)  1.58(0.97-2.56)  2.42(1.11-5.31)  0.008  1  1.49(1.05-1.25)  1.72(1.21-2.48)  2.77(1.98-3.88)  2.99(2.03-4.42)  <0.001 | 0.00  6(8.8%)  23(33.8%)  34(50.0%)  5(7.4%)  4(5.9%)  13(19.2%)  17(25.0%)  19(27.9%)  15(22.1%) | -  1  2.90(1.15-7.29)  6.14(2.46-15.35)  7.81(2.33-26.22)  <0.001  1  1.49(1.05-2.13)  1.72(1.21-4.78)  2.78(1.98-3.88)  2.99(2.04-4.42)  <0.001 | -  1  2.62(1.04-6.65)  4.74(1.89-11.85)  4.24(1.26-14.31)  0.010  1  2.31(0.75-7.10)  3.43(1.14-10.25)  5.93(2.01-17.44)  8.21(2.71-24.84)  <0.001 | 38(15.4%)  66(26.8%)  106(43.1%)  31(12.6%)  5(2.0%)  48(19.5%)  59(23.9%)  48(19.5%)  59(23.9%)  32(13.1%) | 1  0.93(0.62-1.42)  1.42(0.91-2.23)  1.56(0.88-2.77)  5.45(2.38-12.50)  <0.000  1  2.01(0.65-6.27)  2.79(0.92-8.51)  4.25(1.45-12.48)  5.78(1.90-17.58)  <0.001 | 1  0.89(0.59-1.37)  1.19(0.76-1.87)  1.08(0.61-1.91)  2.83(0.97-8.32)  0.055  1  1.48(1.01-2.17)  1.61(1.09-2.40)  2.71(1.88-3.89)  2.71(1.75-4.19)  <0.001 |

^1^ RR are risk ratios (95% Confidence Intervals).

^2^ Adjusted for age, sex, education (none, standard 1-5, standard 6-8, secondary, tertiary), occupation (not working, housework, farming/fishing, self-employed, employed) and wealth status (poorest, 2, 3, 4, wealthiest), as appropriate.

^3^ Overweight defined as BMI 25-29.9 kg/m^2^ and obesity defined as BMI≥30 kg/m^2^.

^4^ Hypertension is defined as SBP ≥140 mmHg, DBP ≥90 mmHg, or self-reported that currently using antihypertensive medication.

^5^ Diabetes mellitus (DM) defined as FBG ≥7.0 mmol/L, or current use of medication prescribed to treat diabetes mellitus or self-reported.

^6^ Multimorbidity defined as the presence of two or more of hypertension, diabetes, and obesity

Suppl.Table 4: Hypertension: Screening, cascade of care, access to diagnostic test, diagnosis, medication and control

|  | Totals | | | Men | | | Women | | |
| --- | --- | --- | --- | --- | --- | --- | --- | --- | --- |
|  | Karonga | Lilongwe | | Karonga | Lilongwe | | Karonga | Lilongwe | |
|  | Rural | Urban | Rural-to-urban migrants | Rural | Urban | Rural-to-urban migrants | Rural | Urban | Rural-to-urban migrants |
| **Of individuals aged ≥40 years N=8,932** | | | | | | | | | |
| Blood pressure measured ever | 1375/5244  26.2% | 675/1070  63.1% | 1814/2618  69.3% | 378/2,181  17·3% | 221/397  55.7% | 632/1040  60.8% | 997/3063  32.6% | 454/673  67.5% | 1182/1578  74.9% |
| Blood pressure measured ≥40 years | 1158/5244  22.1% | 569/1070  53.2% | 1594/2618  60.1% | 350/2181  16.1% | 189/397  47.6% | 563/1040  54.1% | 808/3063  26.4% | 380/673  56.5% | 1031/1578  65.3% |
| **Of overweight and obese individuals N=9334** | | | | | | | | | |
| Blood pressure measured ever | 1311/3109  42.2% | 1616/2321  69.6% | 2776/3904  71.1% | 133/537  24.8% | 212/358  59.2% | 491/723  66.5% | 1178/2572  45.8% | 1404/1963  71.5% | 2295/3181  72.2% |
| **Of all current hypertensives, previously diagnosed and on medication, and undiagnosed N=4,294** | | | | | | | | | |
| Blood pressure measured ever | 697/1888  36.9% | 494/733  67.4% | 1245/1673  74.4% | 214/787  27.2% | 149/286  52.1% | 411/648  63.4% | 483/1101  43.9% | 345/447  77.2% | 834/1025  81.4% |
| Previously diagnosed | 654/1888  34.6% | 317/733  43.3% | 839/1673  50.2% | 172/787  21.9% | 84/286  29.4% | 243/648  37.5% | 482/1101  43.8% | 233/447  52.1% | 596/1025  58.2% |
| On medication | 509/1888  26.9% | 197/733  26.9% | 545/1673  32.6% | 127/787  16.1% | 45/286  15.7% | 152/496  23.5% | 382/1101  34.7% | 152/447  34.1% | 393/1025  38.3% |
| **Of previously diagnosed hypertensives on regular medication and for whom a blood pressure measurement was available N=1245** | | | | | | | | | |
| Blood pressure controlled  <140/90 mmHg | 189/505  37.4% | 77/196  39.3% | 194/544  35.7% | 41/127  32.3% | 17/44  38.6% | 38/152  25.1% | 148/378  39.2% | 60/152  39.5% | 156/392  39.8% |

Suppl.Table 5: Diabetes: Screening, cascade of care, access to diagnostic test, diagnosis, medication and control

|  | Totals | | | Men | | | Women | | |
| --- | --- | --- | --- | --- | --- | --- | --- | --- | --- |
|  | Karonga | Lilongwe | | Karonga | Lilongwe | | Karonga | Lilongwe | |
|  | Rural | Urban | Rural-to-urban migrants | Rural | Urban | Rural-to-urban migrants | Rural | Urban | Rural-to-urban migrants |
| **Of individuals aged ≥40 years N= 8932** | | | | | | | | | |
| Blood glucose measured ever | 249/5244  4.8% | 164/1070  15.3% | 521/2618  19.9% | 109/2181  5.1% | 62/397  15.6% | 209/1040  20.1% | 140/3063  4.6% | 102/673  15.2% | 312/1578  19.8% |
| Blood glucose measured ≥40 years | 228/5244  4.4% | 144/1070  13.4% | 464/2618  17.7% | 99/2181  4.5% | 51/397  12.9% | 188/1040  18.1% | 129/3063  4.2% | 93/673  13.8% | 276/5314  17.5% |
| **Of overweight and obese individuals N=9334** | | | | | | | | | |
| Blood glucose measured ever | 151/3109  4.9% | 259/2321  11.2% | 571/3904  14.6% | 42/537  7.8% | 53/358  14.8% | 166/723  22.9% | 109/2572  4.2% | 206/1963  10.5% | 405/3181  12.7% |
| **Of all individuals with diabetes, previously diagnosed & undiagnosed N=609** | | | | | | | | | |
| Blood glucose test ever | 85/208  40.9% | 64/120  53.3% | 180/281  64.1% | 32/84  38.1% | 26/44  59.1% | 68/101  67.3% | 53/124  42.7 | 38/76  50.0% | 112/180  62.2% |
| Previously diagnosed | 110/208  52.9% | 71/120  59.2% | 177/281  62.9% | 41/84  48.9% | 25/44  56.8% | 67/101  66.3% | 69/124  55.6% | 46/76  60.5% | 110/180  61.11% |
| **Of previously diagnosed diabetics N=358** | | | | | | | | | |
| On regular medication | 80/110  72.7% | 38/71  53.5% | 124/177  70.1%% | 33/41  80.5% | 18/25  72.0% | 42/67  62.7% | 47/69  68.1% | 20/46  43.5% | 82/110  74.6% |
| Blood glucose controlled  FBG <7·0 mmol/L | 53/110  48.2% | 40/71  56.3% | 78/177  44.1% | 21/41  56.8% | 15/25  65.2% | 36/67  59.0% | 32/69  50.8% | 25/46  64.1% | 42/110  40.4% |
| **Of diagnosed diabetic individuals on regular medication N=242** | | | | | | | | | |
| Blood glucose controlled  FBG <7·0 mmol/L | 32/75  40.0% | 15/34  39.5% | 40/119  33.2% | 14/29  48.3% | 9/17  52.9% | 18/39  46.2% | 18/46  39.1% | 6/17  35.3% | 22/80  27.5% |

Suppl.Table 6. Crude and WHO standardised prevalence of overweight/obesity, hypertension and diabetes

|  | Karonga | Lilongwe | |
| --- | --- | --- | --- |
|  | Rural  N=13,903 | Urban  N=6,741 | Rural-to-urban migrants N=9929 |
| **Overweight/obesity**  Crude prevalence  WHO standardized prevalence | 18.4%  19.9% | 30.9%  38.2% | 35.9%  41.3% |
| **Hypertension**  Crude prevalence  WHO standardized prevalence | 13.6%  16.3% | 10.9%  23.4% | 16.9%  26.7% |
| **Diabetes**  Crude prevalence  WHO standardized prevalence | 1.5%  2.1% | 1.8%  5.3% | 2.9%  5.6% |

| **Rural-to-Urban Migrants (N=2512)** | | |
| --- | --- | --- |
| **Reason for migration** | **Total**  **N** | **Percentage**  **%** |
| Marriage | 182 | 7.3 |
| Divorce/Separated | 68 | 2.7 |
| Widowhood | 5 | 0.2 |
| School | 895 | 35.6 |
| Work/employment | 1089 | 43.4 |
| Sickness | 26 | 1.0 |
| Nursing someone | 23 | 0.9 |
| Bereavement | 51 | 2.0 |
| Returning to family | 29 | 1.2 |
| Quarrel | 3 | 0.1 |
| Joining relative | 26 | 1.0 |
| Improving living conditions | 17 | 0.7 |
| Other | 97 | 3.9 |
| Unknown | 1 | 0.0 |

Suppl. Table 7. Reasons for rural-to-urban migration.
